# Modification of the toronto rehabilitation institute—hand function test for integration into robot-assisted therapy: technical validation and usability

**DOI:** 10.1186/s12938-025-01384-7

**Published:** 2025-05-07

**Authors:** Aisha Raji, Stephanie DiNunzio, Andrew Whitmell, Cesar Marquez-Chin, Milos R. Popovic

**Affiliations:** 1 KITE Research Institute, Toronto Rehabilitation Institute, University Health Network, 550 University Ave, Toronto, M5G 2A2 ON Canada; 2 Institute of Biomedical Engineering, University of Toronto, 164 College St, Toronto, M5S 3G9 ON Canada

**Keywords:** Stroke, Spinal cord injury (SCI), Robotic rehabilitation, Upper extremity, 3D printing, Toronto Rehabilitation Institute—Hand Function Test (TRI-HFT), Collaborative robot (cobot), Robot-assisted therapy, Robotic assistance

## Abstract

**Background:**

Effective rehabilitation of the upper extremity function is vital for individuals recovering from stroke or cervical spinal cord injury, as it can enable them to regain independence in daily tasks. While robotic therapy provides precise and consistent motor training, it often lacks the integration of real-world objects that stimulate sensorimotor experiences. The Toronto Rehabilitation Institute—Hand Function Test (TRI-HFT) utilizes 19 everyday items to assess hand function. This study aims to modify the 3D-printed TRI-HFT objects to ensure their compatibility with robotic manipulation, thereby enhancing the functional relevance of robot-assisted rehabilitation, and to evaluate the usability of the new robotic system to ensure its safety and technical performance.

**Results:**

We successfully redesigned the 3D-TRI-HFT objects to enable manipulation by a robotic arm equipped with a gripper. The modified 3D-printed objects closely matched the original specifications, with most weight and size deviations within acceptable limits. Performance tests demonstrated reliable robotic manipulation, achieving a 100% success rate in 50 pick-and-place trials for each object without any breakage or slippage. Usability assessments further supported the system’s performance, indicating that participants found the system engaging, useful, and comfortable.

**Conclusions:**

The modified 3D-printed TRI-HFT objects allow seamless integration into robotic therapy, facilitating the use of real-world objects in rehabilitation exercises. These modifications enhance functional engagement without compromising user interaction with the objects, demonstrating the feasibility of combining traditional rehabilitation tools with robotic systems, potentially leading to improved outcomes in upper extremity rehabilitation. Future research may focus on adapting these designs for compatibility with a broader range of robotic equipment, reducing the cost of the objects as 3D printing technology advances, and evaluating the system’s performance among individuals with stroke and SCI.

**Supplementary Information:**

The online version contains supplementary material available at 10.1186/s12938-025-01384-7.

## Background

Restoring voluntary movement of the upper extremity is crucial for individuals with hemiplegia following stroke or cervical spinal cord injury (cSCI), as it enables them to maintain independence in activities of daily living (ADLs) [1–5]. Rehabilitation plays an important role in reducing residual motor deficits resulting from upper extremity impairments in these populations [6]. Current approaches recommend treatments that are intensive, highly repetitive, and task-oriented to promote functional recovery of upper extremity functions [3, 5, 7, 8]. Therapeutic interventions of the upper extremity primarily focus on restoring reaching and grasping abilities, which are fundamental for the functional use of the hands and arms [3, 9]. Achieving such functional recovery is crucial for assisting individuals within these populations to regain independence and improve quality of life [5]. Hence, rehabilitation interventions often prioritize functional goals.

Robotic rehabilitation offers the capability to provide intensive, repetitive, and task-specific motor training, while also delivering precise, prolonged, and consistent interventions [5, 10]. This approach leads to significant time and effort savings for patients and therapists [10]. Additionally, robots can objectively measure outcomes and automate repetitive tasks, enhancing the efficiency of therapy sessions [11].

However, current robotic devices predominantly deliver therapy through game-like activities displayed on monitors [12]. While these virtual tasks can be engaging, they may not fully replicate the sensorimotor experiences associated with real-life object manipulation [13]. To enhance the effectiveness of robot-assisted therapy, it is important to incorporate real activities that stimulate sensorimotor input by using functional objects during training [6, 7, 14]. By focusing on functional tasks that mirror daily activities, robot-assisted therapy can better support patients in regaining independence and reintegrating into social and domestic life [11, 14].

**Fig. 1:**
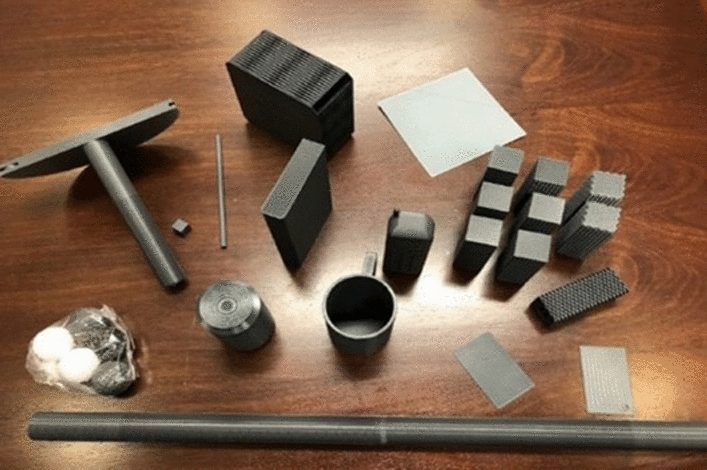
Items in the 3D-printed Toronto Rehabilitation Institute—Hand Function Test [16]

The Toronto Rehabilitation Institute—Hand Function Test (TRI-HFT) is a comprehensive assessment tool designed to evaluate power grasp (palmar grasp) and precision grip (lateral pinch and pulp pinch) [15]. The test consists of two parts: object manipulation and strength measurement. The object manipulation portion of the test involves the use of everyday objects that require different grips. As shown in Fig. 1 and Table 1, the 3D-printed test items include a mug, a sheet of paper, a book, a reusable plastic bag filled with golf balls, a soda can, a die, a sponge, a credit card, a wireless home telephone, a pencil, and nine rectangular blocks categorized in sets of 3$\times$100 g, 3$\times$200 g, and 3$\times$300 g, each with surfaces of varying friction levels (smooth, medium, and rough) [16]. The strength measurement component of the test assesses the strength of lateral grip and palmar grasp through three sub-tests involving an instrumented cylinder, an instrumented credit card, and a graduated wooden bar.

To promote the practice of diverse grasp patterns and functional tasks during robot-assisted therapy, we propose incorporating items in the object manipulation part of the TRI-HFT into the therapeutic process. We expect this integration to encourage the use of various grasp patterns and enhance the performance of functional activities. However, enabling a robotic arm to manipulate these objects effectively without obstructions during therapy sessions is challenging. Therefore, the objective of this paper is to present the modifications made to the TRI-HFT items, specifically those used for object manipulation, to facilitate exercise during upper extremity robotic therapy while maintaining their assessment properties of upper extremity function.

## Results

### Modification of objects in the Toronto Rehabilitation Institute Hand Function Test

**Fig. 2:**
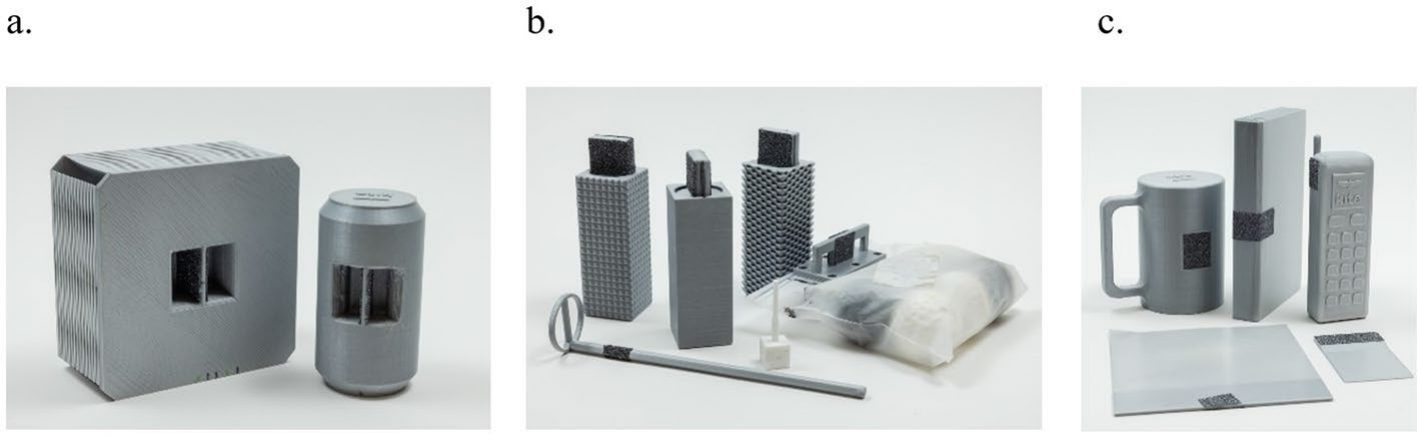
The modified 3D TRI-HFT items with **a** internal handles, **b** external handles, and **c** handleless designs

We successfully 3D-printed the modified objects with internal handles, external handles, and handleless designs, as presented in Fig. 2. The dimensions of these modifications are detailed in App﻿endix A﻿. Except for the sponge and paper, we replicated the physical dimensions of all objects within a tolerance of 0.1 mm. Our weight measurements revealed a maximum error margin of 5% compared to the original TRI-HFT objects; however, the phone, pencil, credit card, and mug exhibited weight variations of up to 10%. Detailed measurements for the modified printed objects are provided in Table 1.

**Table 1:** Dimensions of the original TRI-HFT, the 3D-printed TRI-HFT, and the modified 3D-printed TRI-HFT objects [16]

No.	Object	Measurement of original TRI-HFT objects (size in mm, weight in g)	Measurement of 3D-printed TRI-HFT objects (mm, g)	Measurement of modified 3D- printed TRI-HFT objects (mm, g)
1	Mug	Size: 115 $\times$ 245	Size: 115 $\times$ 250	Size: 115 $\times$ 156
		Weight: 563.98	Weight: 554	Weight: 545
2	Paper	Size: 297 $\times$ 210 $\times$ 0.1	Size: 150 $\times$ 150 $\times$ 0.3	Size: 150 $\times$ 150 $\times$ 0.3
		Weight: 0	Weight: 8	Weight: 14
3	Book	Size: 173 $\times$ 105 $\times$ 26	Size: 173 $\times$ 105 $\times$ 26	Size: 173 $\times$ 105 $\times$ 26
		Weight: 315	Weight: 318	Weight: 316
4	Pencil	Size: 187 $\times$ 5.9	Size: 190 $\times$ 7	Size: 198 $\times$ 6
		Weight: 6	Weight: 6	Weight: 7
5	Zip lock bag	Size: 170 $\times$ 200	Size: 170 $\times$ 200	Size: 170 $\times$ 200
	With golf balls	Weight: 230 (46g per ball	Weight: 236 (39g per ball	Weight: 228 (38 per ball
		$\times$ 5 golf balls)	$\times$ 6 golf balls)	$\times$ 5 golf balls)
6	Sponge	Size: Isosceles triangle with	Size: Square 142 $\times$ 142	Size: Square 142 $\times$ 142
		height 400 and base 200		
		Weight: 170	Weight: 161	Weight: 178
7	Die	Size: 15 $\times$ 15 $\times$ 15	Size: 16 $\times$ 16 $\times$ 16	Size: 16 $\times$ 16 $\times$ 16
		Weight: 6	Weight: 4	Weight: 5
8	Rectangular blocks	Size: 115 $\times$ 35 $\times$ 35	Size: 100 $\times$ 36 $\times$ 36	Size: 115 $\times$ 38 $\times$ 38
		Weight: 100/200/300	Weight: 100/200/296	Weight: 100/200/300
9	Wireless phone	Size: 144 $\times$ 50 $\times$ 25	Size: 145 $\times$ 50 $\times$ 35	Size: 145 $\times$ 50 $\times$ 36
		Weight: 223	Weight: 222.3	Weight: 215
10	Soda can	Size: 120 $\times$ 61	Size: 123 $\times$ 66	Size: 123 $\times$ 66
		Weight: 350	Weight: 388	Weight: 360
11	Credit card	Size: 85 $\times$ 53	Size: 86 $\times$ 54	Size: 86 $\times$ 54
		Weight: 0	Weight: 4	Weight: 3

### Support shelf

**Fig. 3:**
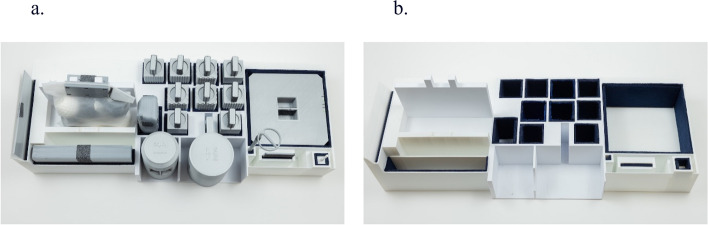
The support shelf** a**) with objects** b**) without objects

We fabricated a support shelf using 3D printing, resulting in an empty weight of 1.72 kg (see Appendix B for dimensions). Figure 3 displays the shelf with and without the modified objects. The support shelf was used to ensure consistent placement of the objects for the cobot to retrieve and return.

### Object gripping and manipulation

**Fig. 4:**
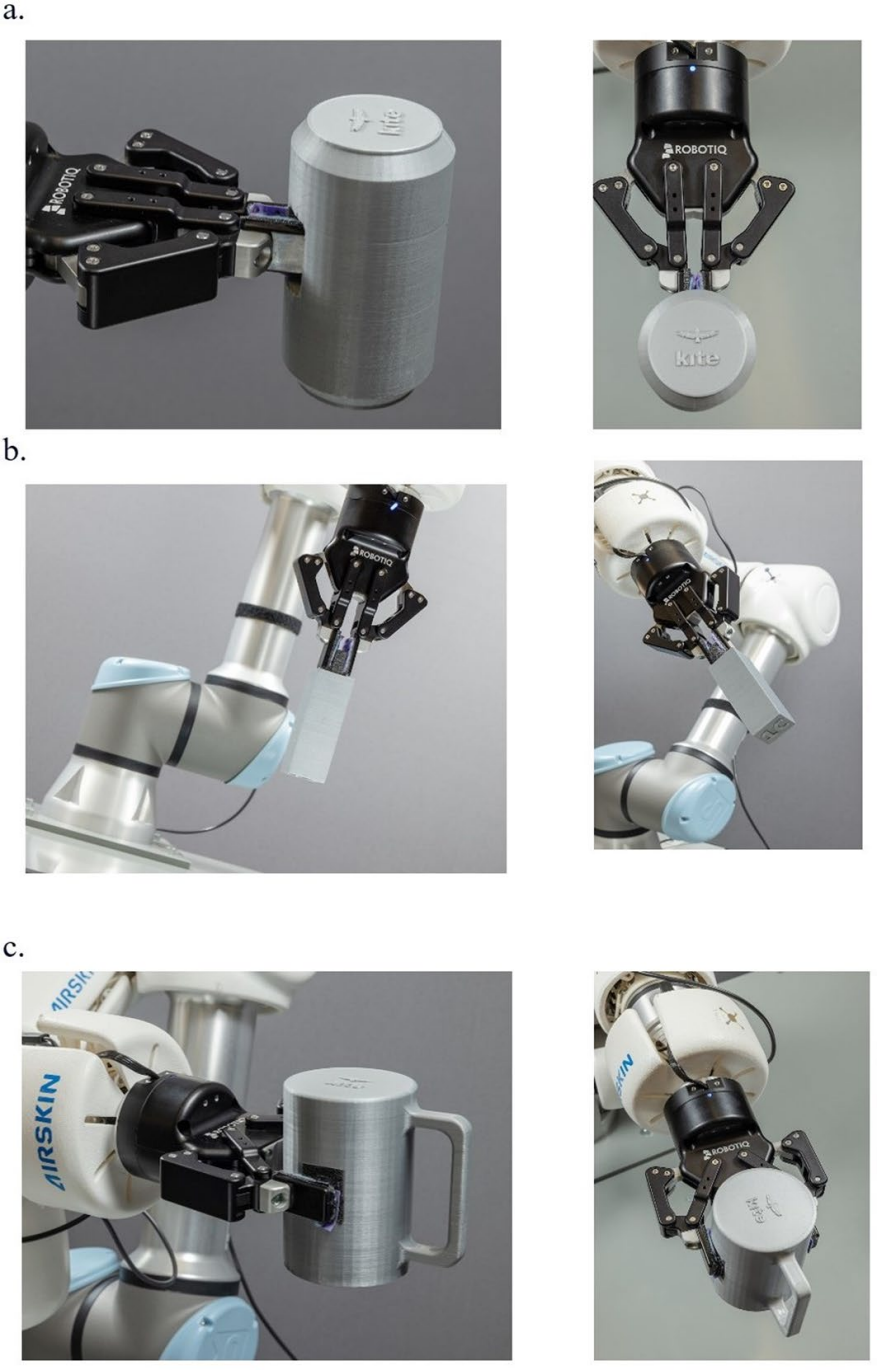
The end-effector gripping objects with **a** internal handle (side and top views) presented at 180$^{\circ }$, **b** external handle (front view) presented at 90$^{\circ }$ and 45$^{\circ }$, and **c** handless design (side and top views) presented at 180$^{\circ }$

We evaluated the performance of the modified objects using the 2F-85 adaptive gripper (Robotiq, Canada) attached to the cobot described in the Methods section. We applied non-slip rubber coverings to the gripper’s fingertips to enhance friction between the object handles and the gripper. As shown in Fig. 4, we tested the robot’s ability to manipulate the modified objects at three different orientations (45$^{\circ }$, 90$^{\circ }$, and 180$^{\circ }$). We conducted 50 pick-and-place trials to assess the breakage and slippage of objects from the gripper. In all trials, the robot successfully picked up and returned all objects from the support shelf without breakage or slippage (i.e., 100% success).

### Testing with participants

#### Participants

We recruited five participants with normal upper extremity function, no neurological conditions, and no prior experience with robotic rehabilitation in a single 3-h session to evaluate the usability and preliminary results of the system. As shown in Table 2, 60% of the participants were men and 40% were women, 19 to 72 years (mean age = 48.8 years). No adverse event was reported during the study.

**Table 2:** Demographic information of participants

Participant ID	Age	Gender
P001	32	M
P002	19	F
P003	70	F
P004	72	M
P005	51	M

#### Usability evaluation of the system

i. Qualitative feedback

**Fig. 5:**
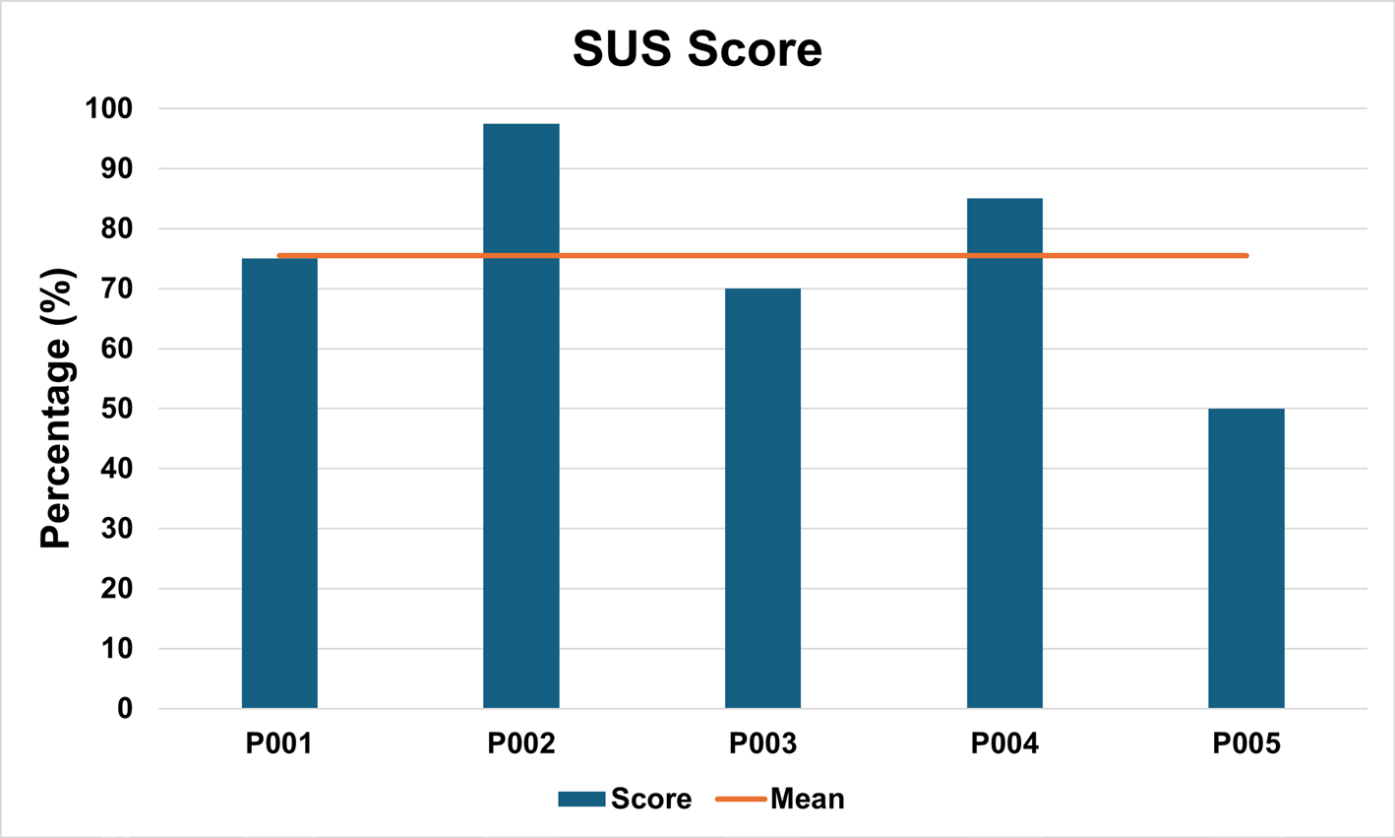
Individual results on the SUS

We collected qualitative data using the System Usability Scale (SUS), the Intrinsic Motivation Inventory (IMI), and open-ended questions to determine user acceptance and experience. Participants rated the system’s usability with an overall mean of 75.5% (SD = 17.1), as shown in Fig. 5. For the SUS, a usability score of 68% or higher is considered ‘Good’, according to Bangor et al. [17].

**Fig. 6:**
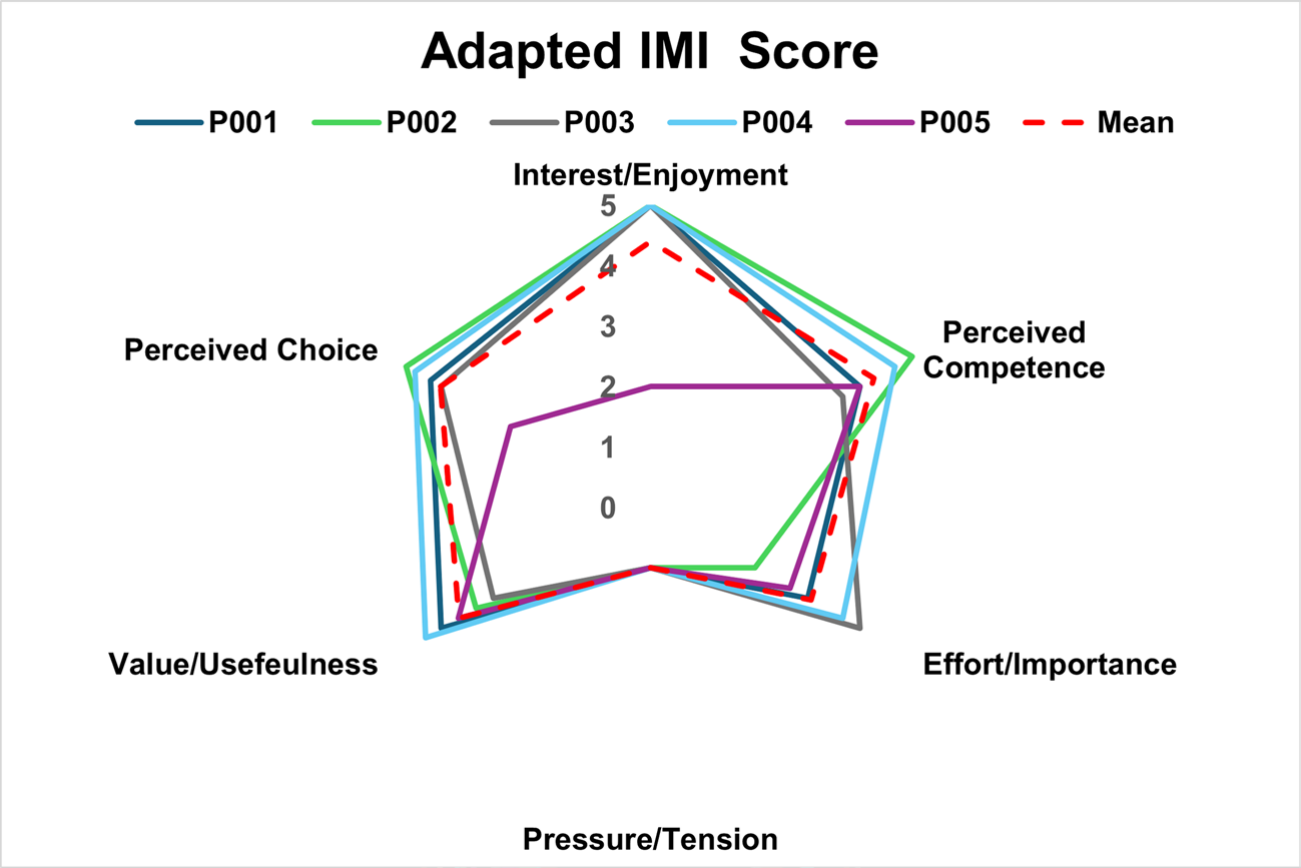
Individual results on the adapted IMI scale

The IMI data (Fig. 6) indicated that participants reported high level of interest and enjoyment (4.40 ± 1.34), felt competent (4.27 ± 0.55), and did not feel pressured while performing the tasks (1.00 ± 0.00). They also rated their effort levels positively (3.07 ± 0.80), found the tasks useful (3.66 ± 0.52), and indicated they had voluntary control over the system (4.01 ± 0.79).

Participants provided positive feedback regarding the usage of the system. They highlighted specific aspects they found engaging, such as the diversity of grasps required, the variety of objects the system could handle, the challenge mode (described in the Methods section), and the innovative reach assessment. For example, one participant stated, “I enjoyed the different grasps needed”, while another commented, “I enjoyed the extent of reach where I had to spin the robot and move it vertically”. However, some participants identified areas of improvement. Noting that “the rough block felt rigid to grasp” and that “the robot operated at a slow speed”.

ii. Quantitative feedback

We evaluated participant’s performance during the extent of reach exploration and object reaching and grasping tasks.

-* Phase 1* extent-of-reach assessment

**Fig. 7:**
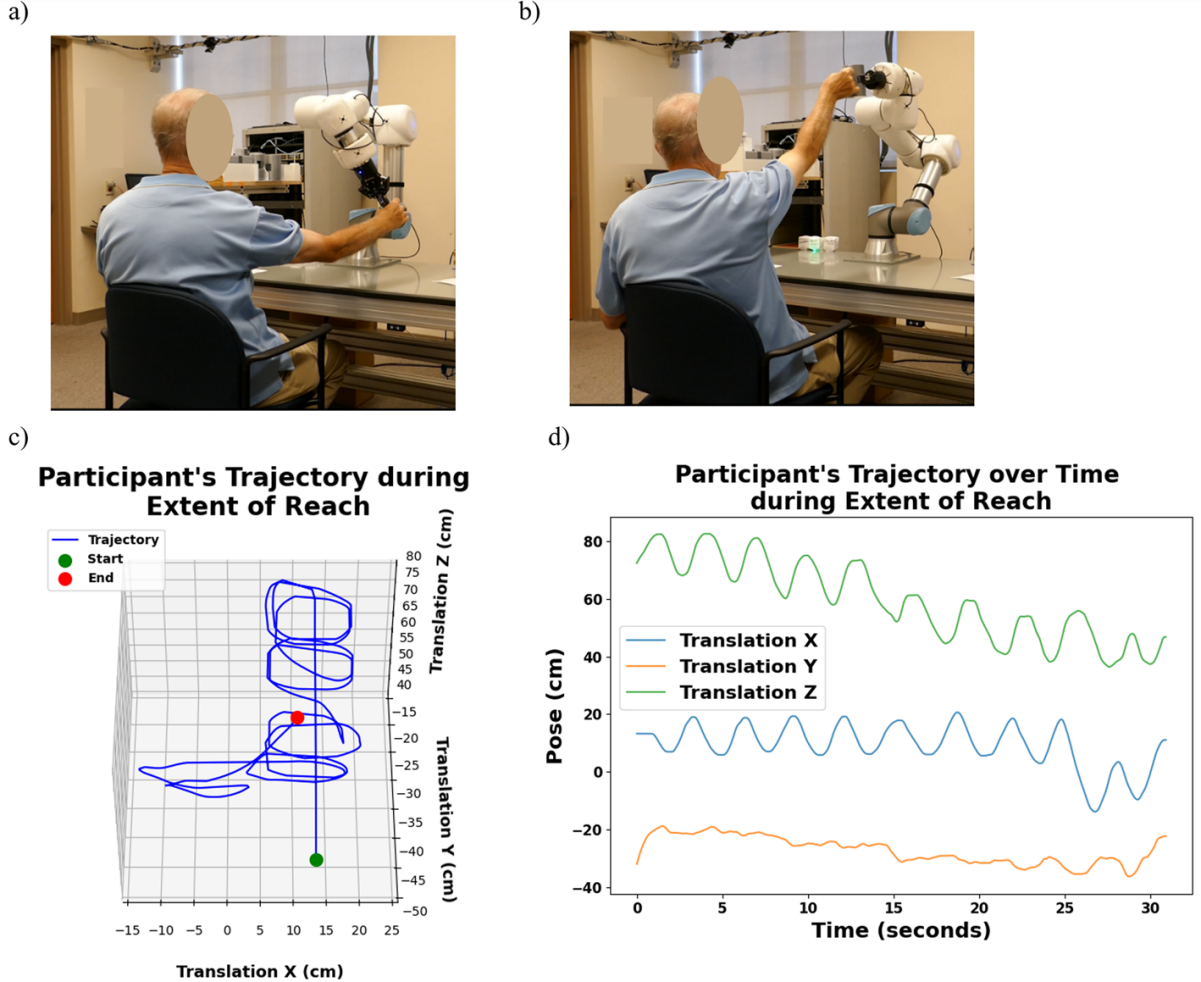
**a** A participant moving object during extent-of-reach assessment; **b** participant grasping mug during the challenge mode; **c** 3D visualization of the recorded trajectories for the participant during the extent-of-reach assessment; **d** 2D representation of reach in various axes during extent-of-reach assessment for the participant

The table (150 x 75 cm) provides the spatial context for our analysis. Figure 7a shows a participant performing the extent-of-reach assessment task, while Fig. 7b shows the participant executing the reach and grasp task. T trajectory (Fig. 7c) highlights the three-dimensional spatial pathway with the start and end points, indicating that the participant produced defined, symmetrical spirals. Participants completed the assessment in a mean of 33.33 s (Fig. 7d), achieving a mean range of 53.56 cm along the x-axis, with a maximum of range of 86.86 cm—consistent with normative data for horizontal reach values (80–100 cm) [18–20]. Along the y-axis, participants reached a mean range of 33.42 cm (maximum 42.08 cm), and along the z-axis, a mean of 52.12 cm (maximum 56.48 cm). Table 3 presents the translation values recorded during extent-of-reach assessment, with mean and standard deviation values highlighting variability across all axes.

**Table 3:** Position recorded in various axes during extent-of-reach assessment

Position axis	Range (cm)	Mean ± SD (cm)
X	86.86	60.25 ± 0.54
Y	32.15	16.88 ± 0.65
Z	54.75	25.85 ± 0.97

**Fig. 8:**
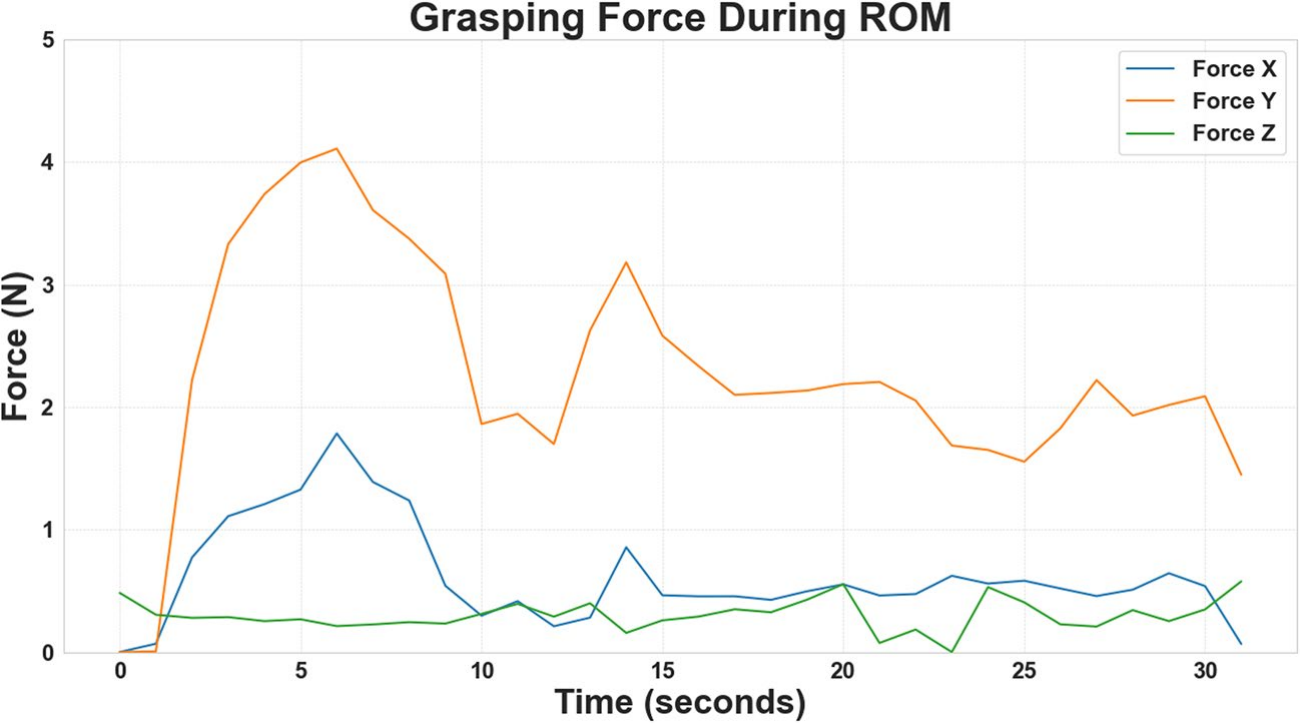
The grasping force exerted in various axes during extent-of-reach assessment for the participant

The force variability recorded during the extent-of-reach assessment is presented in Fig. 8 and detailed in Table 4. The participant generated a total force of 6.47 N, with mean forces of 0.62 N, 2.28 N, and 0.381 N on the x-, y-, and z-axes, respectively. Additional graphs for the remaining participants are provided in Appendix C.

**Table 4:** Forces recorded in various axes during extent-of-reach assessment

Force axis	Range (N)	Mean ± SD (N)
X	1.78	0.62 ± 0.01
Y	4.11	2.28 ± 0.04
Z	0.58	0.38 ± 0.01
Total	6.47	3.28 ± 0.05

-* Phase 2* object reaching and grasping task

**Fig. 9:**
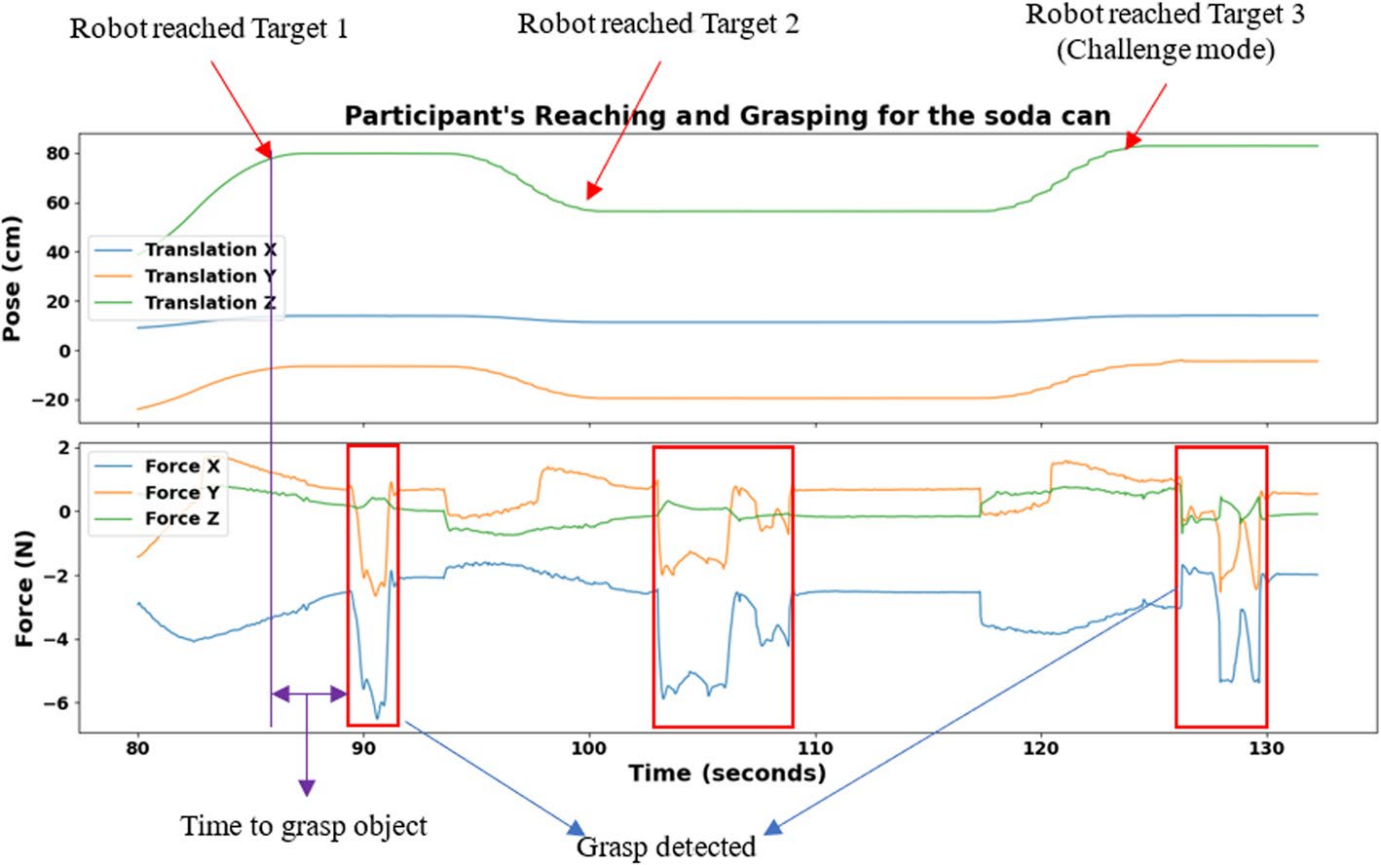
Target position and force detection during the reach-and-grasp task with the soda can

Fig. 9 shows the position (X, Y, and Z) and force data for a participant during three distinct reach-and-grasp cycles with a soda can. Each cycle involves different target placement in 3D space and varying force levels. The position profiles illustrate how the robot places the soda can within or beyond (target 3) the participant’s reach, while the force profiles indicate the participant’s grasp force on the soda can at each target.

**Fig. 10:**
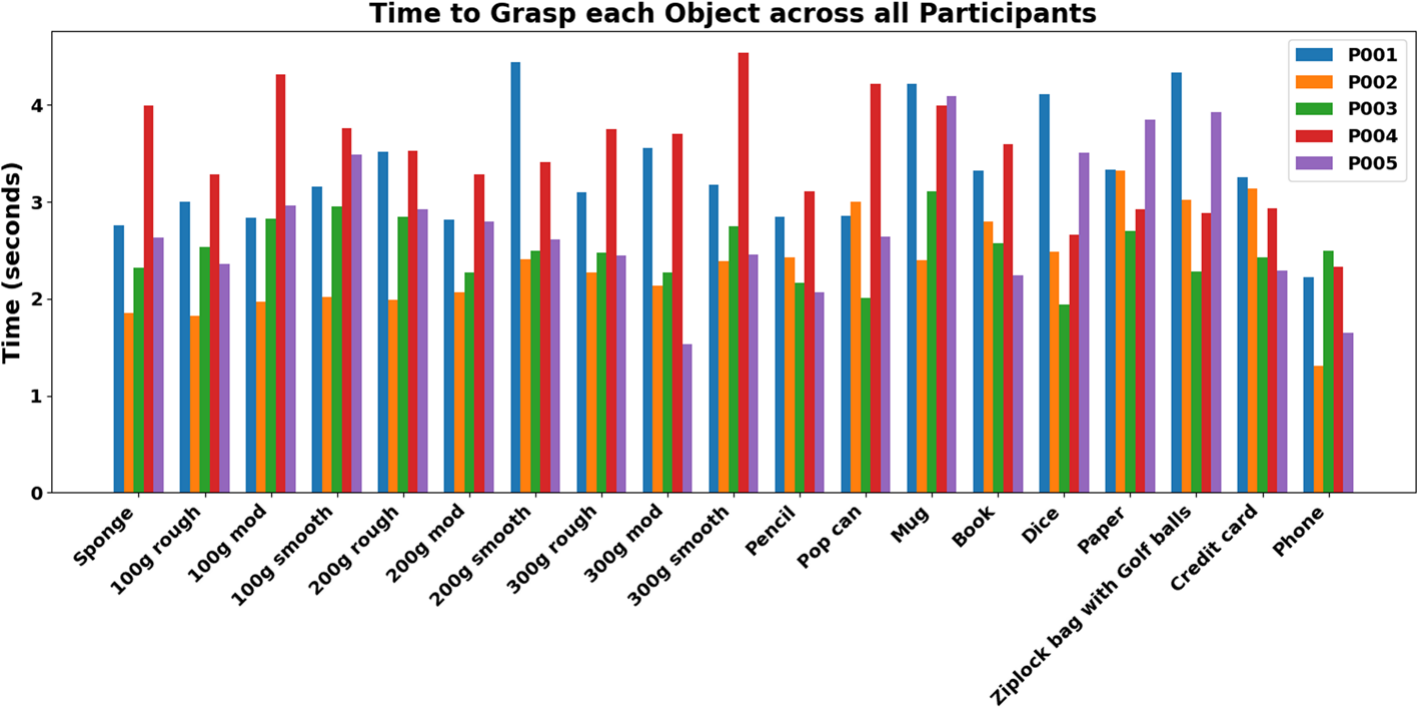
Time to grasp each object by participants

**Fig. 11:**
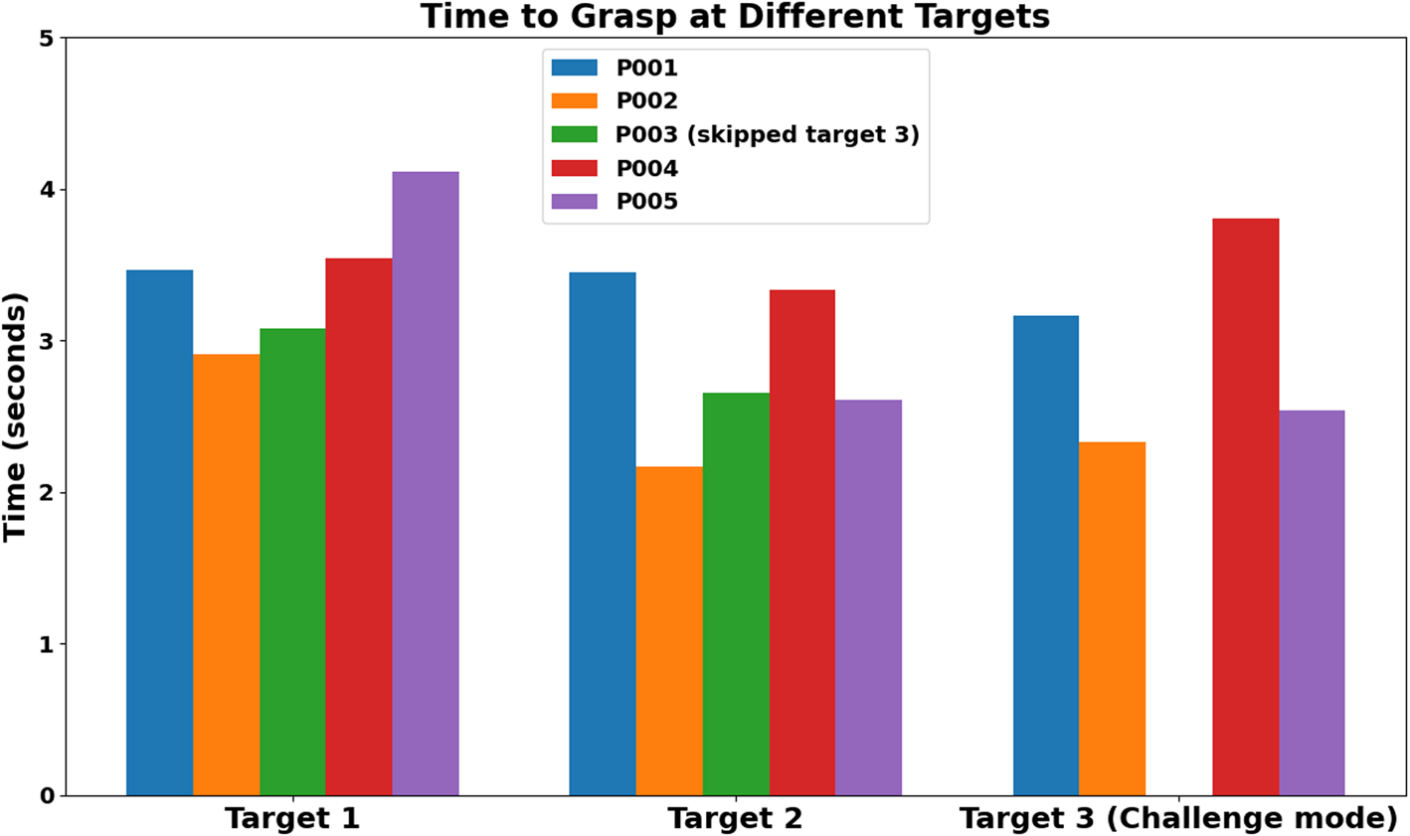
Time to grasp at each target during the object reaching and grasping task

The results show variations in the time taken to grasp objects across both participants and object types, as illustrated in Figs. 10 and 11. Figure 10 presents the reach times for each participant across all objects, with an overall mean of 3.08 s. For example, objects such as the phone and pencil required a shorter time to grasp, whereas heavier items like the mug and a resealable plastic bag (Ziploc®, S.C. Johnson & Son, Inc., U.S.A.) containing golf balls took slightly longer. Figure 11 displays that the time taken to grasp objects ranged from 2.17 to 4.11 s, with noticeable differences among participants. Some individuals maintained consistent time to grasp across objects, while others showed greater variability, suggesting differences in hand function, dexterity, or grasp strategies.

Analysis of individual performance (Fig. 11) shows that participant P001 displayed similar times to grasp across all targets. Participant P002 exhibited the fastest time to grasp, with a reduction from target 1 to target 2 and a slight increase at target 3, potentially due to the target position being outside the participant’s reach extent. The time to grasp for participant P003 improved from target 1 to target 2, but did not participate in the challenge mode (target 3). Participant P004 maintained a steady time to grasp between target 1 and target 2 with a slight increase at target 3. They also exhibited relatively high time to grasp at target 2 and target 3, compared to all other participants. Participant P005 had the slowest time to grasp at target 1 but improved from target 1 to target 2, maintaining a similar time to grasp at target 3.

Participants P002, P003, and P005 showed improvements in time to grasp across all targets, suggesting the participants’ adaptability and improved efficiency through learning and practice. In contrast, participants P001 and P004 demonstrated a more stable time to grasp, possibly indicating a performance limit or the influence of factors such as fatigue. The longer time to grasp at the first target may reflect initial unfamiliarity with the objects and their presentation. These findings highlight the impact of individual differences and object-specific characteristics on motor performance during reaching and grasping tasks.

**Fig. 12:**
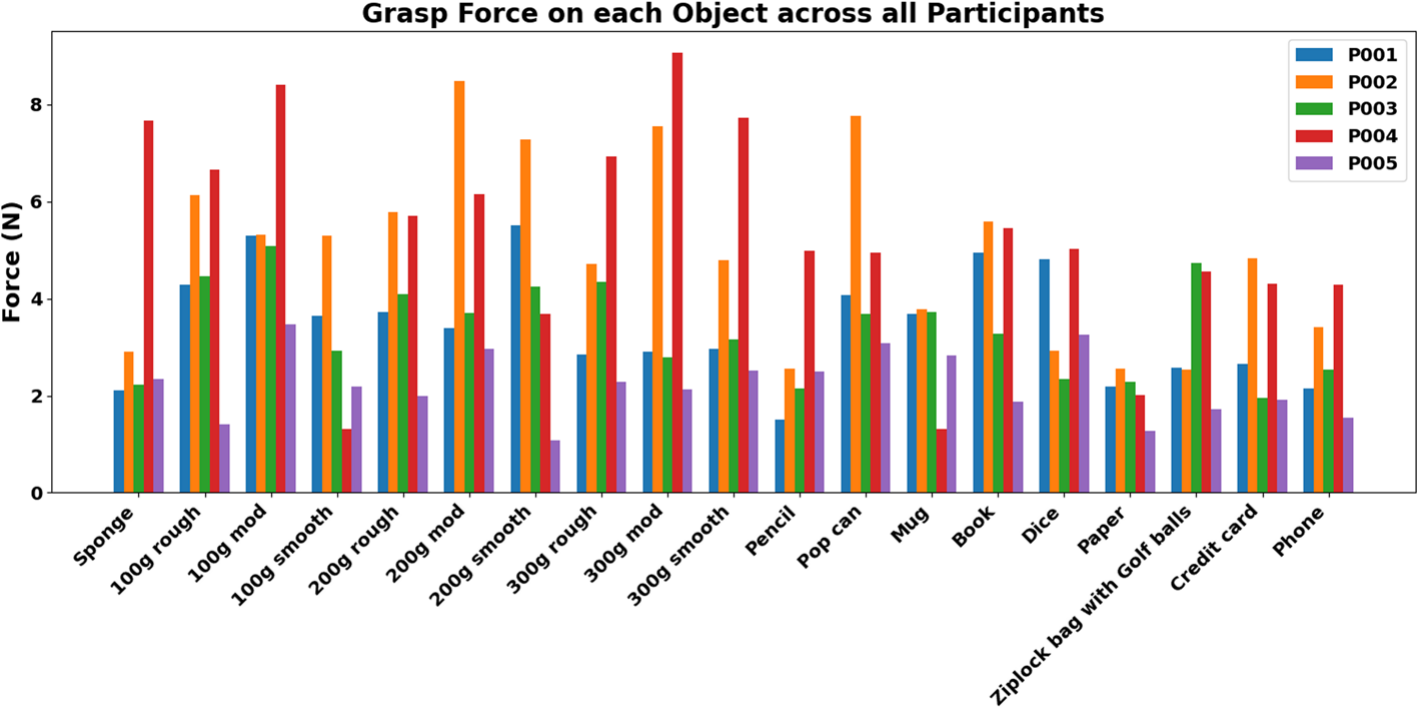
Time to grasp each object by participants

**Fig. 13:**
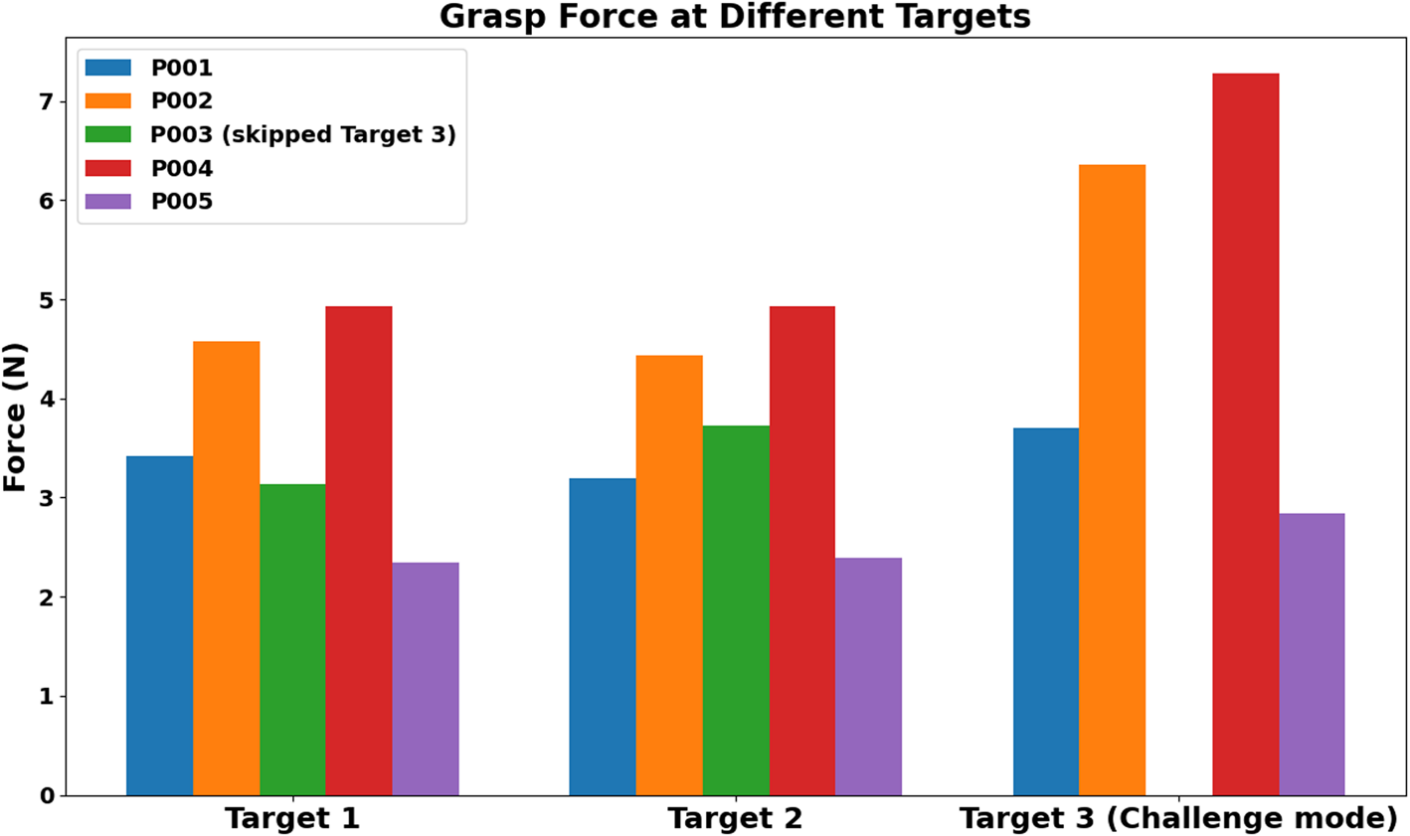
Time to grasp at each target during object reaching and grasping task

The grasp force across all objects (Fig. 12) also shows distinct patterns across participants and objects. The force generally increased during the challenging task (Fig. 13), potentially reflecting the more demanding target position.

Each participant exhibited a unique force profile, suggesting individual differences in grip strength or grasp strategies. Participants P002 and P004 consistently applied higher forces across most objects, while P003 and P005 used relatively lower forces.

Analysis of individual trials indicates that participants P002 and P004 demonstrated high forces across all objects (Fig. 13), with a notable increase in target 3. Participant P001 showed moderate grasp force overall but exhibited an increase in the challenge mode, suggesting potential learning or adaptation. Participant P005 maintained relatively low forces with a slight increase at target 3. In contrast, participant P003 demonstrated moderate forces in targets 1 and 2, but direct comparison was limited by the absence of data at the third target. Overall, these findings highlight the interaction between participant-specific factors and object characteristics in grasp force during reaching and grasping tasks.

## Discussion

The aim of this study was to modify the 3D-printed TRI-HFT objects for integration into a novel robotic rehabilitation approach and evaluate the usability of this method for upper extremity rehabilitation to ensure its safety and technical performance. The modifications have helped us develop a new paradigm in robotic rehabilitation.

The original 3D-printed TRI-HFT objects, fabricated in our Rehabilitation Engineering Lab at the KITE Research Institute, were modified using existing STEP files obtained from the KITE Research Institute, University Health Network, Toronto, Canada [16]. The redesigned objects were fabricated with high dimensional accuracy, achieving a tolerance of 0.1 mm for most objects. Some objects presented printing challenges that necessitated dividing them into multiple parts which were later combined to obtain the final object. For example, we redrew the sponge into six parts and assembled them after printing. Size and weight measurements showed a maximum error of 5% compared to the original TRI-HFT, but some items, such as the phone, pencil, credit card, and mug, varied in weight by up to 10%. These variations may be due to inconsistencies in material density or manufacturing processes and may affect the gripper’s performance, especially in tasks requiring precise weight distribution and delicate handling. Future research should address these sources of weight variation and explore adjustments in material selection or manufacturing techniques to ensure uniformity across all objects.

The 3D-printed support shelf provided a stable platform for object placement and ensured a consistent object arrangement and location during testing. This shelf contributed significantly to the system’s reliability by supporting the systematic organization of objects during the pick-and-place tasks.

Our modifications resulted in a design that is both functional and reproducible for robotic manipulation. The UR5e cobot, equipped with the 2F-85 adaptive gripper whose fingertips were enhanced with rubber coverings to increase friction, executed 50 pick-and-place trials. During testing, difficulties emerged in manipulating the phone and book with the robotic arm, primarily due to the positioning of the handles, located farther from the object’s center of gravity. This initial approach involved grasping these objects by their originally designed internal handles (see Appendix A). However, this method proved inefficient because of the imbalance caused by the off-center handles. To resolve this challenge, we adjusted the pickup approach by grasping the objects from their sides, which improved handling efficiency and stability during manipulation. The system achieved a 100% success rate.

It is important to emphasize that these designs are specifically tailored for use with the 2F-85 adaptive gripper. Therefore, users intending to use these modified TRI-HFT objects should use the same or a dimensionally equivalent gripper to achieve similar results, as the current designs may not be universally compatible with all grippers.

Quantitative performance metrics further validate the technical performance of the system. During the extent-of-reach assessment, participants completed the task in a mean of 33.33 s. Reach ranges along the x-, y-, and z-axes closely aligned with normative data. The variability in the recorded data demonstrates the system’s sensitivity to variations in motor performance and capturing individual differences in reach-and-grasp tasks.

Variations in time taken to grasp and forces across objects and participants may indicate distinct motor profiles and grasp strategies. For example, objects such as the phone and pencil required shorter time to grasp, while heavier items like the mug and resealable plastic bag containing golf balls took longer. Faster time taken to grasp may indicate higher motor control and adaptation, while longer time to grasp may suggest a need for additional training. Differences in force application also reflect individual variations in grasp strength, strategy, and task familiarity. This highlights the system sensitivity to individual motor performance.

Object-specific characteristics such as surface texture may also influence force application, as suggested by the higher forces recorded across the rough and moderate blocks compared to the smooth blocks, although more data are needed to confirm this observation.

Usability metrics further support the technical performance of the system. Participants rated the system as engaging, useful, and comfortable, as evidenced by a mean SUS score of 75.5% and positive IMI ratings. Qualitative feedback highlighted the advantages of diverse grasp types, the challenge mode, and the innovative reach assessment while also recommending improvements in the texture of the rough block and the operational speed of the robot.

These findings highlight the successful advancement of the TRI-HFT design, demonstrating the effectiveness of our modifications in achieving broader compatibility and enhanced ease of use for robotic rehabilitation of the upper extremity. The optimized designs have proven effective in the current setup, with all objects being successfully picked up, manipulated, and returned to the shelf by the robotic arm. Future work may explore adaptations of these designs for compatibility with a wider range of robotic grippers and further cost reductions as 3D printing technologies evolve.

## Conclusions

In this paper, we presented the modifications made to the 3D-printed TRI-HFT designed to enable robotic manipulation during upper extremity rehabilitation. By redesigning the objects to facilitate easy pickup, manipulation, and return by a robotic arm, we achieved seamless execution of the test in a clinical setting. These modifications support reliable robotic performance without compromising the user’s ability to interact with the objects, potentially enhancing both the efficiency of the test and the rehabilitation process. The system demonstrated high dimensional accuracy, effective object handling, and positive feedback from individuals with normal upper extremity function and no neurological conditions. Our work demonstrates that integrating real-world objects into robot-assisted therapy is feasible, paving the way for more functional and engaging rehabilitation protocols. Future research will expand the clinical application of the system among individuals with stroke and SCI to further evaluate its performance and usability.

## Methods

### Modification of objects in the Toronto Rehabilitation Institute Hand Function Test

We modified all the objects in the 3D-printed TRI-HFT to ensure compatibility with the robot gripper. We excluded objects used for strength measurement (instrumented cylinder, instrumented credit card, and wooden bar) because they are not part of the object manipulation portion of the test. We implemented these modifications using 3D CAD software (SolidWorks 2022, Dassault Systèmes, San Diego, CA, USA). The original 3D models of the objects were provided by the KITE Research Institute in the Standard for the Exchange of Product Data (STEP) file format, a widely used standard for sharing detailed 3D geometry across different CAD systems [16].

During the modification process, we ensured user safety by avoiding sharp edges and maintaining unobstructed object manipulation. We designed the modifications to minimize changes in how users manipulate the objects while ensuring they could be easily grasped by the gripper. We categorized the object modifications into three main designs: internal handle, external handle, and handleless.

#### Internal handle designs

For the internal handle design, we added internal pockets within the objects to allow the robot’s gripper to reach inside and securely grasp them. Specifically, we redesigned the soda can and sponge using this approach. We carefully selected suitable locations on each object to provide sufficient space for the gripper to fit inside. We added two pockets around a handle within each object, enabling the gripper to clamp onto it effectively. The pocket dimensions are 17 mm by 35 mm for the soda can and 20 mm by 35 mm for the sponge. Due to the complexity of the sponge’s structure, we divided it into six component parts before assembling. This design preserved the original shape and size of the objects while minimizing any impact on the user’s ability to manipulate them. Furthermore, it allowed users to grasp the objects from any orientation since the gripper did not make contact with the exterior surfaces of the objects.

#### External handle designs

For objects in this category, we added external handles separate from the main body of the object to enable effective grasping by the gripper. We modified the die, pencil, golf ball holder, and rectangular blocks with external handles, each requiring unique design solutions different from the internal handle approach. For the die, we extruded a thin handle from one face to facilitate easier user grasping and manipulation. For the pencil, we added a handle on one end, allowing users to manipulate it from the opposite end while preventing it from rolling when placed on a flat surface. We redesigned the golf balls bag by adding a separate handle attached to the reusable plastic bag, allowing the balls to move freely within the bag while still being easily manipulable. Finally, we left the main body of the rectangular blocks unchanged but modified the screw-on caps to include an extruding handle for robot gripping and manipulation. This modification preserved the different textures on the block faces while maintaining the integrity of the original testing parameters.

#### Handleless designs

We made small modifications to the bodies of certain objects without adding separate handles to allow direct clamping by the robot gripper. The objects redesigned without handles include the mug, credit card, and paper. We modified the mug to include a flat section on each side, enabling the gripper to hold it by its side walls. This design encouraged users to grasp the mug by its handle rather than the sides, given the positioning of the gripper. Similarly, we altered the credit card and paper by slightly thickening one end to facilitate secure holding by the gripper without risking breakage. Despite these modifications, we preserved the primary function of these objects and avoided introducing stress points that could cause the objects to break.

#### 3D printing of the modified objects

We 3D-printed the modified objects using an Ultimaker S5 (Ultimaker, The Netherlands) printer with a 0.4-mm AA print nozzle and polylactic acid (PLA) material. We used a fine printing resolution of 0.1 mm (3.94 thou) for all objects, adjusting the infill according to each object’s mass requirements. We covered the handles with silicon carbide traction tape to enhance the friction between the objects and the gripper. We added Canadian coins to the printed blocks to achieve the original rectangular blocks’ weight. For the 100 g blocks, we included two 5¢ coins (each weighing 3.95g), two 10¢ coins (each weighing 1.75g), and two 25¢ coins (each weighing 4.4g). The 200 g blocks, included one 5¢ coin (weighing 3.95g), one 10¢ coin (each weighing 1.75g), and fifteen 25¢ coins (each weighing 4.4g). Finally, for the 300 g blocks, we included four 5¢ coins (each weighing 3.95g) and forty 25¢ coins (each weighing 4.4g).

### Support shelf

We designed a storage shelf to facilitate easy access to the modified objects and ensure their consistent location for the robot. Using SolidWorks, we developed several shelf design prototypes, systematically exploring vertical and horizontal placement styles. This process involved testing shelf positions at the front, sides, and back of the workspace and optimizing placements to minimize the robot’s travel time. We selected the horizontal placement style because it consistently reduced travel time and avoided robot singularities. The final design has 19 compartments; each dimensioned to hold a specific object and 3D-printed with PLA material. Due to the printer’s build volume limitations, we printed the shelf in three separate parts and assembled them to form the complete unit. We lined the interior of each compartment with felt material to prevent scratching and ensure a secure fit. We further reinforced the compartments designated for the rough blocks with velvet lining to prevent the felt from degrading with regular use. We strategically positioned the shelf on a platform attached to the robot’s table.

### Object gripping and manipulation

#### Robot

In this study, we used a UR5e robotic arm (Universal Robots, Odense, Denmark). The UR5e is a collaborative robot (cobot) with six degrees of freedom, which allows for high flexibility and precision in movement, making it suitable for tasks that require complex manipulation. It has a payload capacity of 5 kg and a reach of 850 mm, providing a balance between reach and strength for handling the objects used in this study. Additionally, the arm has force-sensing capabilities that enable safe interaction with humans and the environment, making it ideal for settings that require precise and safe manipulation of objects, as required in this study.

#### End-effector

We equipped the cobot arm with an end-effector, the 2F-85 gripper (Robotiq Inc., Québec, Canada). The 2F-85 is designed for robotic applications that require versatility in grasping different objects. It has two parallel fingers, each 22 mm wide, with a maximum stroke of 85 mm, allowing it to securely grasp a wide variety of objects with different sizes and shapes. The gripper’s adjustable force and speed parameters make it suitable for both delicate and firm gripping tasks. This adaptability and precision make it well-suited for use in various manipulation tasks within our experimental setup.

### Testing with participants

#### Participants

The intervention involved individuals with normal upper extremity function and no neurological conditions who participated in a single 3-h exercise session to assess the safety and usability of the system. Ethics approval was obtained from the Research Ethics Board at the University Health Network in Toronto, Canada, and all participants provided written informed consent. These initial evaluations served as a preliminary step, and future testing with individuals with stroke and SCI to validate the device’s effectiveness and usability in the intended population.

- Testing Procedure

The robot was placed on a table and participants sat on a chair positioned at a safe and comfortable distance from the table’s edge. We provided participants with clear instructions and expectations, including a video demonstration to familiarize them with the procedure. We then configured the robot by selecting the affected upper extremity to be used throughout the session. An experimental session consisted of two phases (described next), in which the participant performed a series of tasks following visual cues.

i. * Phase 1* 3D extent-of-reach assessment

In the first phase of the rehabilitation session, we conducted a one-time extent-of-reach assessment to evaluate each participant’s three-dimensional workspace (range of movement (ROM)). This assessment identified the maximum and comfortable reach limits across various axes, which were used to guide the subsequent phase of the session. The robot presented a rectangular block at the center of the table’s edge without releasing it. When a “start exploration” visual indicator was illuminated, we asked if the participants were comfortable with the block’s position and adjusted it if necessary.

Participants then freely used their chosen upper extremity to move the block vertically as high as possible on the z-axis, and gradually draw the largest spiral possible while gradually moving the block down towards the table surface. This movement captured their maximum reach in the x-, y-, and z-axes (lateral, anterior, and vertical). Figure 7 illustrates the setup and data recorded during this process. After the exploration, the “start exploration” indicator illuminated again, and the robot waited 10 s to ensure that the user had released their grasp before returning the block to the shelf.

ii. * Phase 2* object reaching and grasping task

In the second phase, the system randomly selected and presented objects for a reach-and-grasp task at two random positions within each participant’s unique movement range, identified in the first phase. When the “grasp object” indicator illuminated, the participant reached, grasped, and pulled the object until the indicator deactivated. The indicator turned off when a force threshold of 5N was exceeded or after one minute (if the participant could not reach the target position).

We also implemented a challenge mode to encourage participants to extend their reach beyond the measured range of movement. After the “grasp object” indicator deactivated at the second target position, the “challenge mode” indicator illuminated, prompting the participant to accept the challenge. If the participant accepted, the robot moved the object 5 cm outside the previously determined workspace. The “grasp object” indicator illuminated again at the challenge position, prompting the participant to grasp and pull the object. Once the indicator deactivated, the robot dropped off the object and returned to its default home position.

#### Usability evaluation of the system

i. Qualitative feedback

We assessed the usability of the system and user motivation using the System Usability Scale (SUS) [21] and the Adapted Intrinsic Motivation Inventory (IMI) [22]. The SUS measures usability on a scale from 0 to 100%, with a threshold of 68% considered acceptable.

We used the IMI scale, which is a multidimensional measurement method used to assess participants’ subjective experiences during the task. The full IMI consists of 45 items across seven subscales, this study used an adapted version with 20 items. These items were grouped into six subscales: interest/enjoyment, perceived competence, effort, pressure/tension, perceived choice and value/usefulness. Participants rated each statement on a Likert scale ranging from ‘strongly agree’ to ‘strongly disagree’. Furthermore, we collected short-answer responses regarding their experiences, including features they liked, disliked, and suggestions for improvement.

We analyzed the qualitative data using Minitab©software (Minitab, LLC, U.S.A.). We calculated the mean and standard deviation for the Likert-scale items in the SUS and Adapted IMI. Qualitative responses were processed using data from the open-ended questions and analyzed using thematic analysis.

ii. Quantitative feedback

We collected the data with a frequency of 500 samples per second and applied a low-pass filter to them with a cutoff frequency of 1 Hz to align with the frequency range of human motion [23, 24]. To evaluate the ROM, we rescaled the data using equation (1) and evaluated both the range of reach and force exerted on the object on each axis:

1 $$x_{{{\text{normalized}}}} = \frac{{x - \sigma }}{{\sqrt n }},$$

where $n$ is the number of data points recorded and $\sigma$ is the standard deviation of the data.

In the second phase, the robot used the data from the first phase to calculate the maximal reaching workspace by selecting the outer edge of the path and the area of the workspace. We evaluated the mean time to grasp an object as the interval between the grasp cue and the participant’s actual grasp, and we assessed the mean force exerted on each object as the force measured between the object grasp and release along each axis. The standard error of the mean (SEM) was calculated using equation (2):

2 $$\begin{aligned} \text {SEM} = {\sigma }/{\sqrt{n}}. \end{aligned}$$

## Supplementary Information

Supplementary file 1.

Supplementary file 2.

Supplementary file 3.

## Data Availability

Not applicable.

## Abbreviations

3D: Three-dimensional
ADLs: Activities of daily living
cSCI: Cervical spinal cord injury
Cobots: Collaborative robots
DOFs: Degrees of freedom
PLA: Polylactic acid
STEP: Standard for the Exchange of Product Data
TRI-HFT: Toronto Rehabilitation Institute—Hand Function Test
UR: Universal Robots
