# Supplementary material for: Modification of the toronto rehabilitation institute—hand function test for integration into robot-assisted therapy: technical validation and usability
Source: Biomed Eng Online. 2025 May 7;24:54. doi: 10.1186/s12938-025-01384-7 (PMC12060526; doi:10.1186/s12938-025-01384-7)
Supplement: Supplementary file 1 [file 12938_2025_1384_MOESM1_ESM.docx]

**Appendix A: Dimensions of the Modifications Made to the TRI-HFT Objects in mm**

| 1. **Objects with internal handles** | |
| --- | --- |
|  | 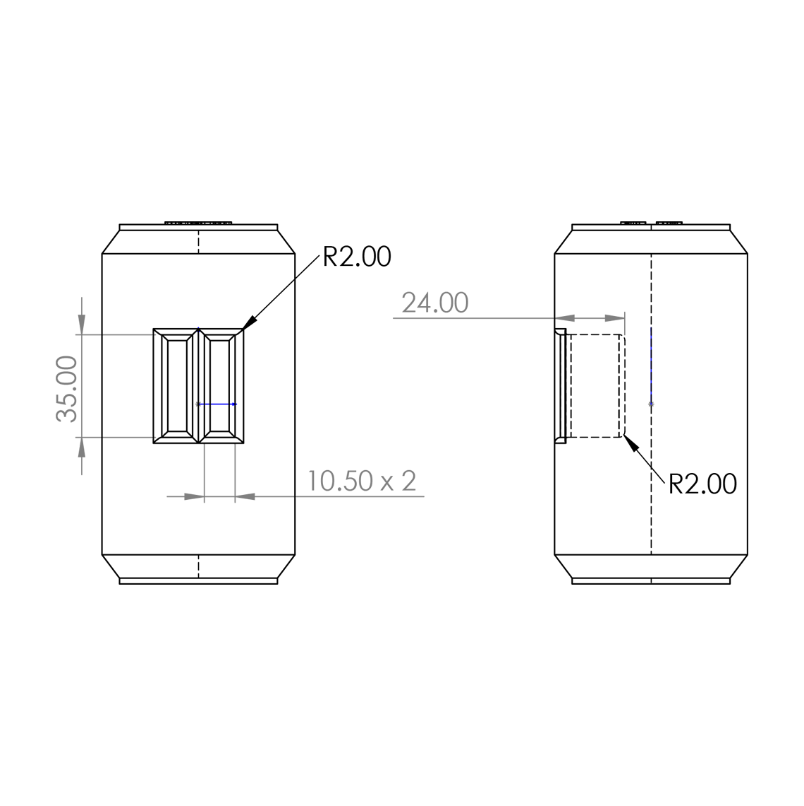 |
|  | 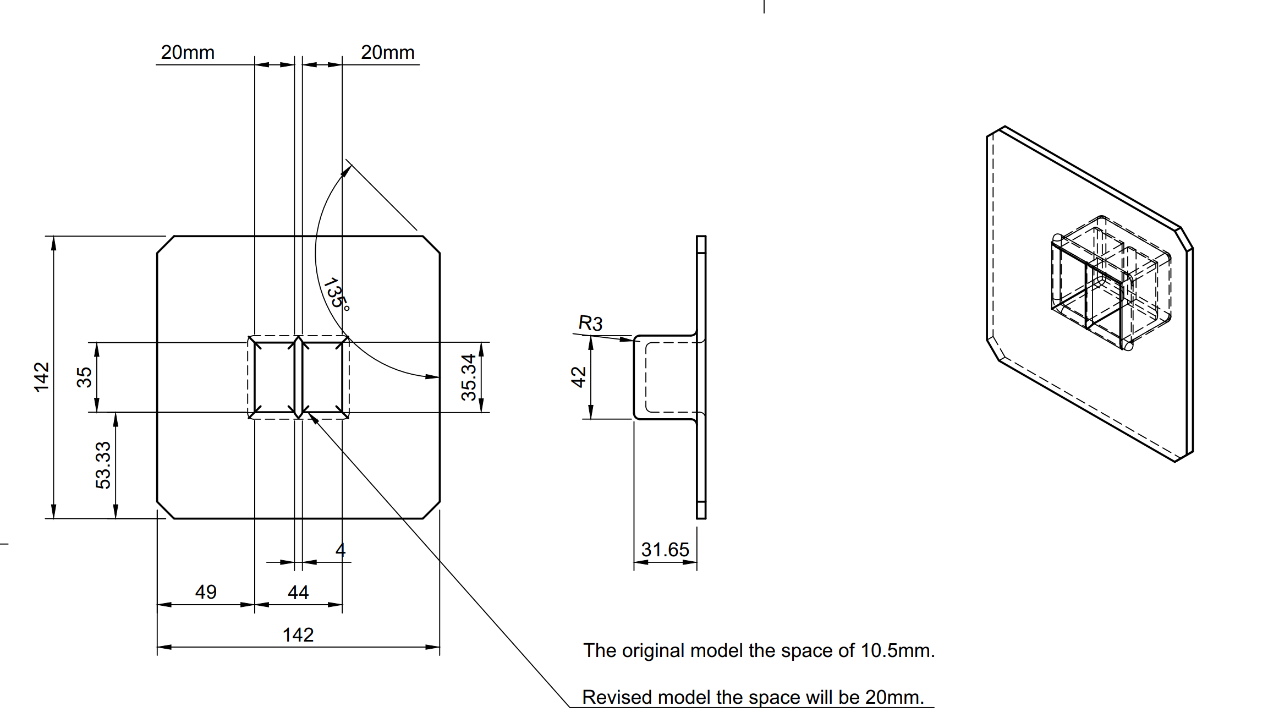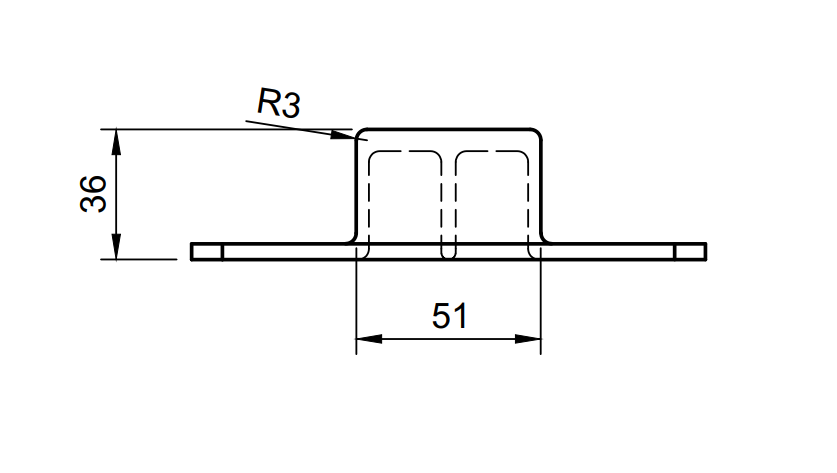 |
|  | a) Soda can b) Sponge |

| 1. **Objects with external handles** | | |
| --- | --- | --- |
|  | 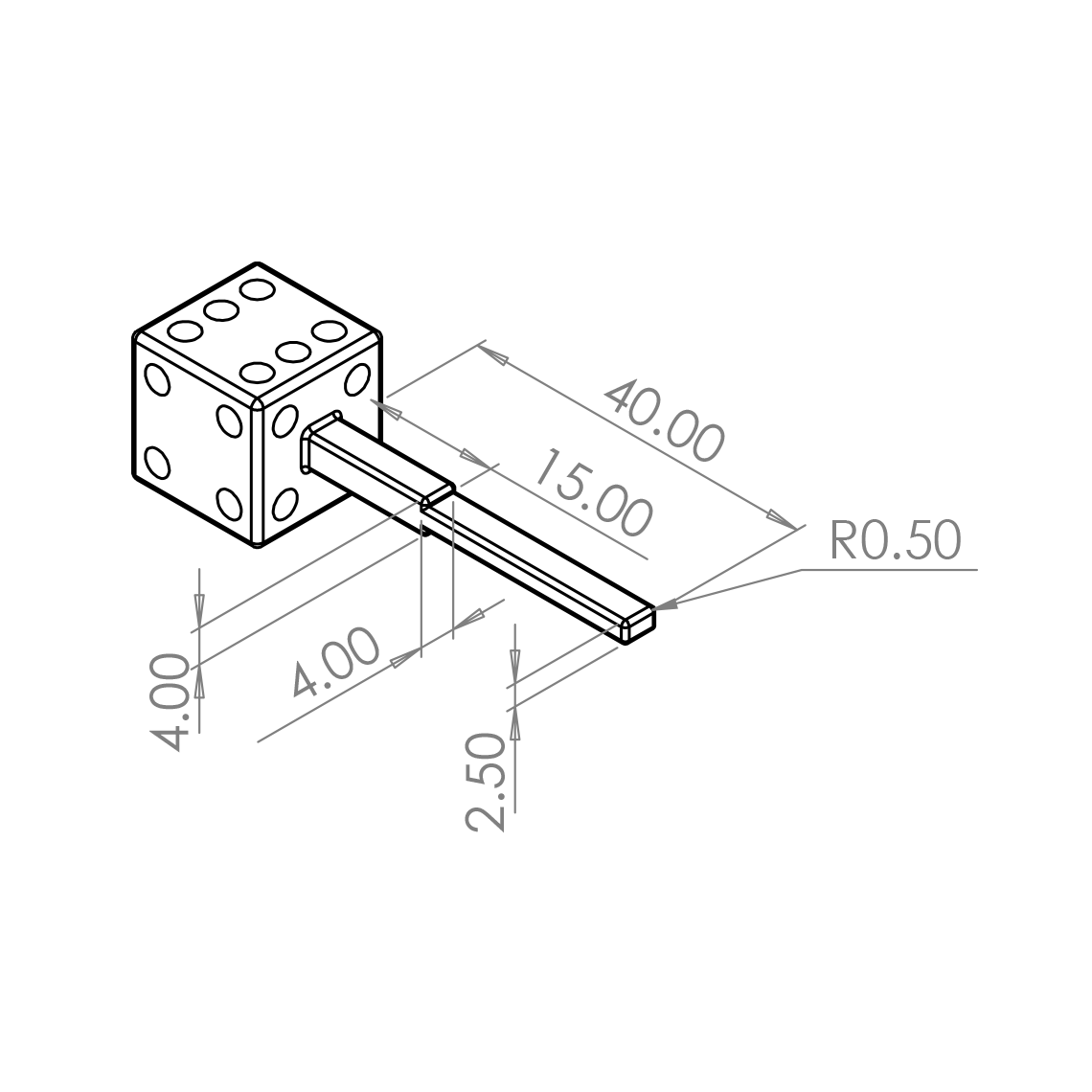 | 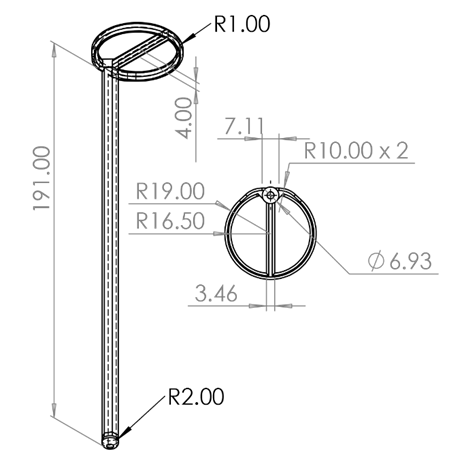b. |
| c. | 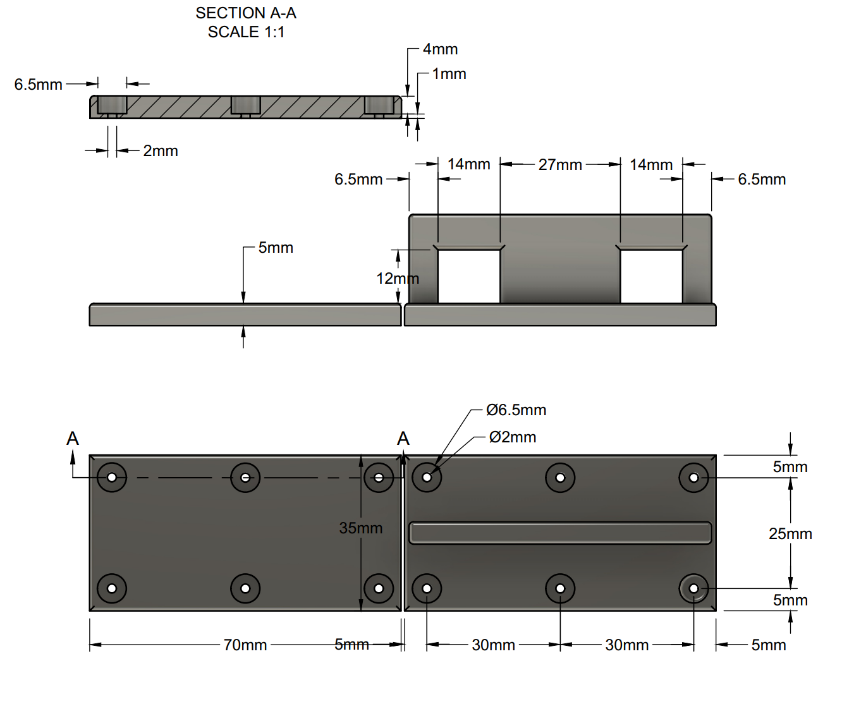 | 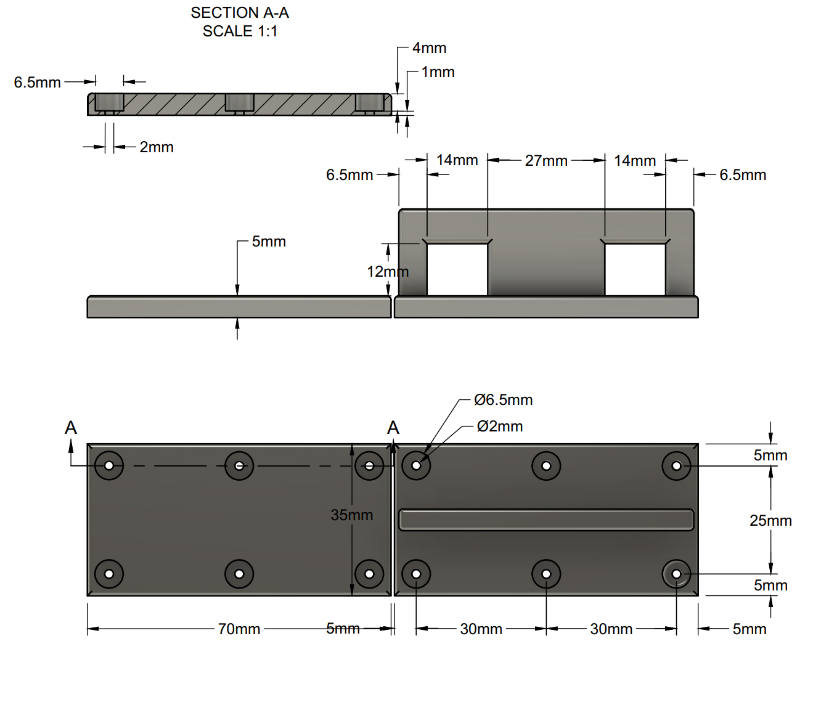 |
| d. | 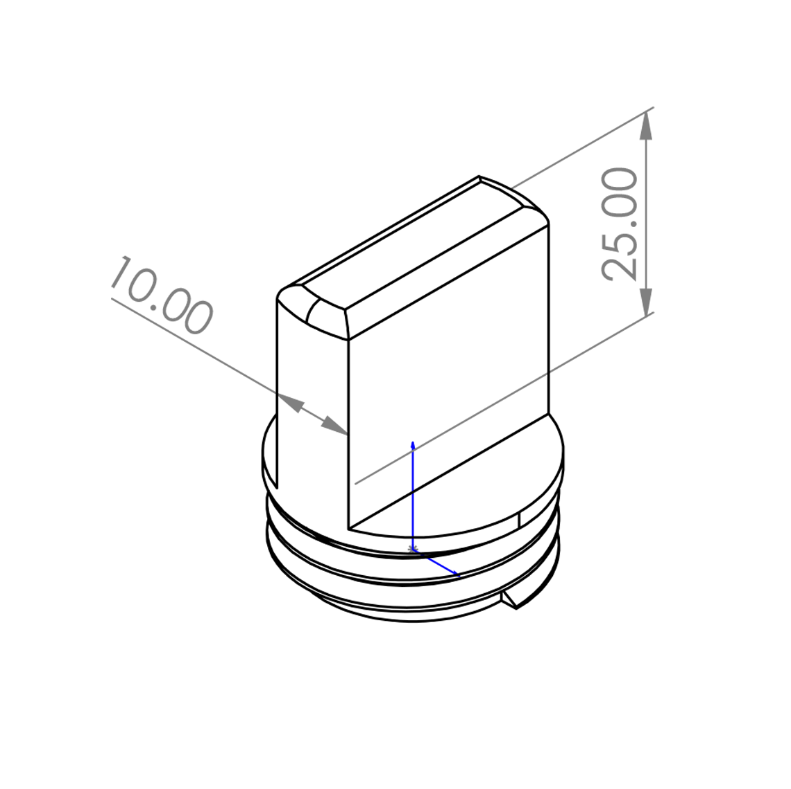 |  |
| a) Rolling die b) Pencil c) Golf ball holder d) Rectangular block | | |

| 1. **Handleless Objects** | | |
| --- | --- | --- |
|  | 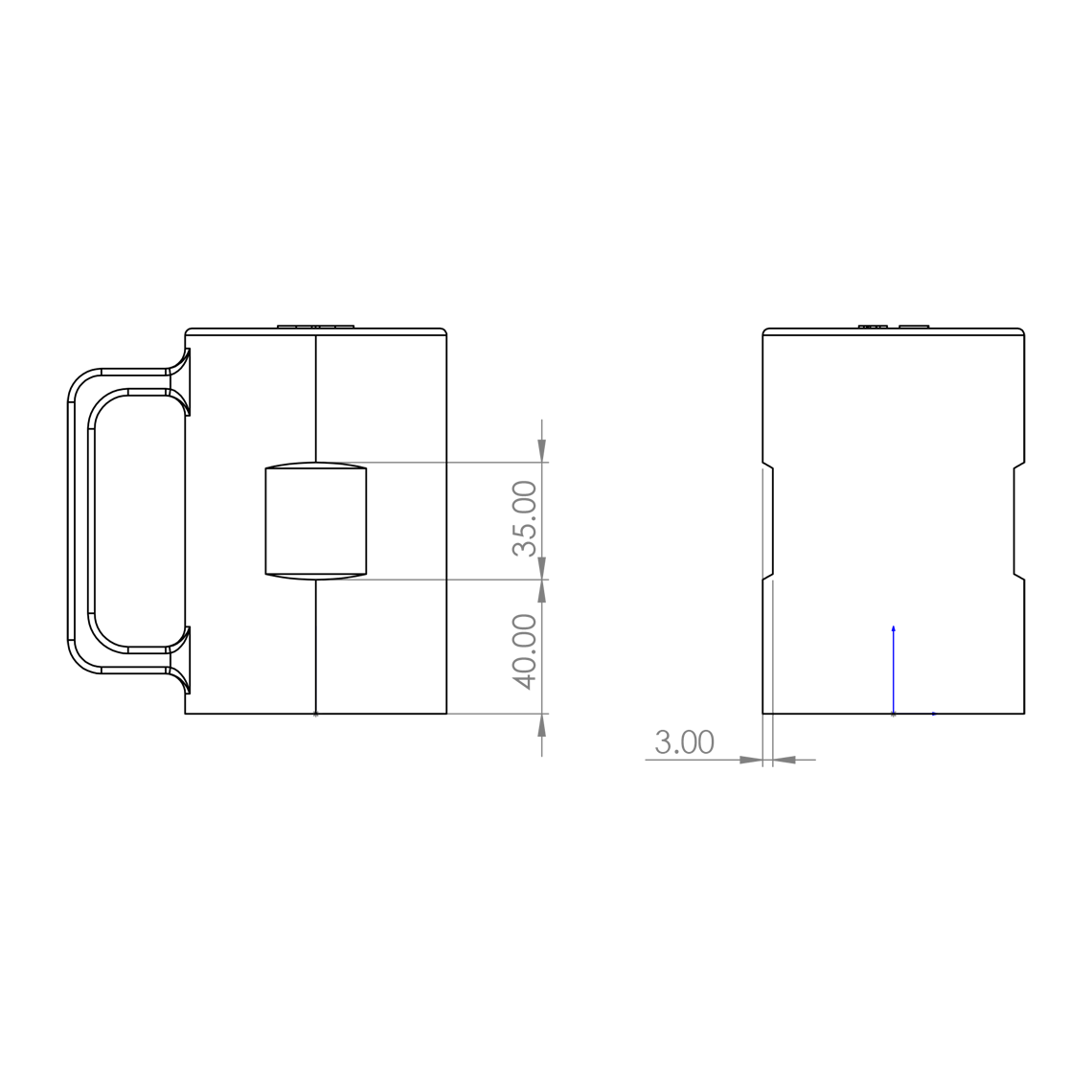 | |
|  | 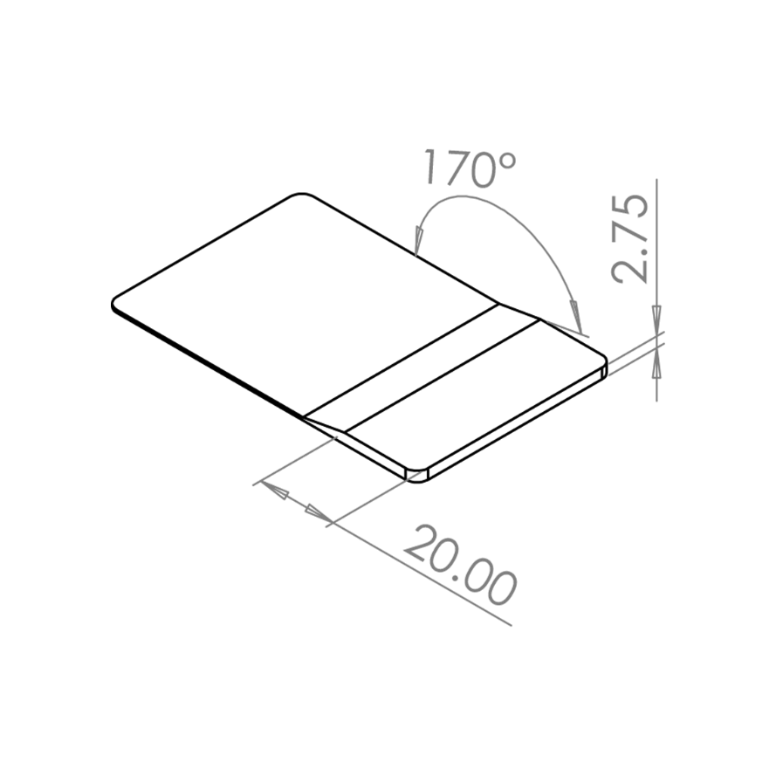 | 1. 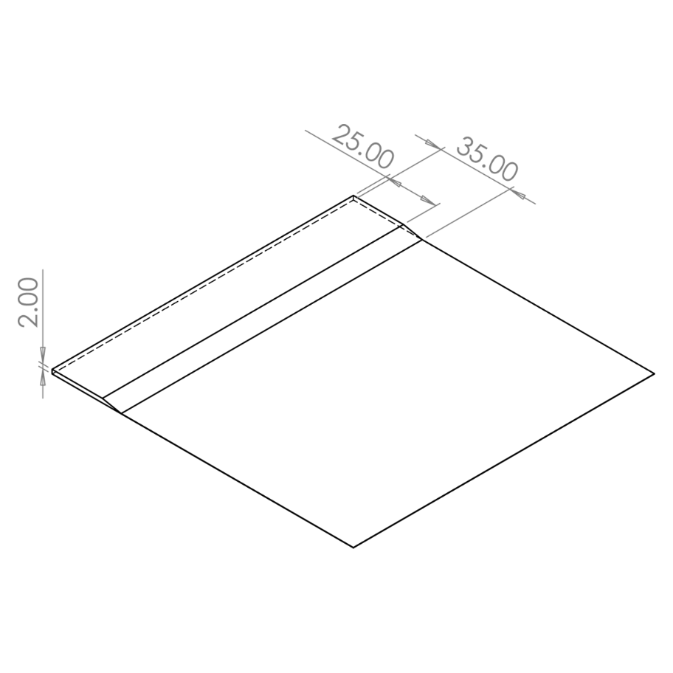 |
| a) Mug b) Credit card c) Paper | | |

| 1. **Original design for the wireless phone and book** | |
| --- | --- |
|  | 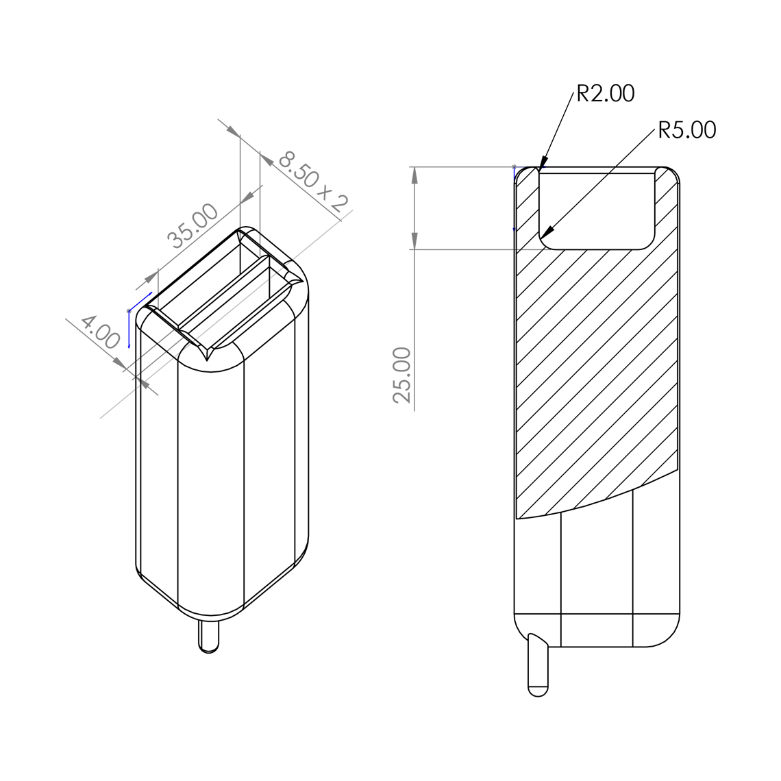 |
|  | 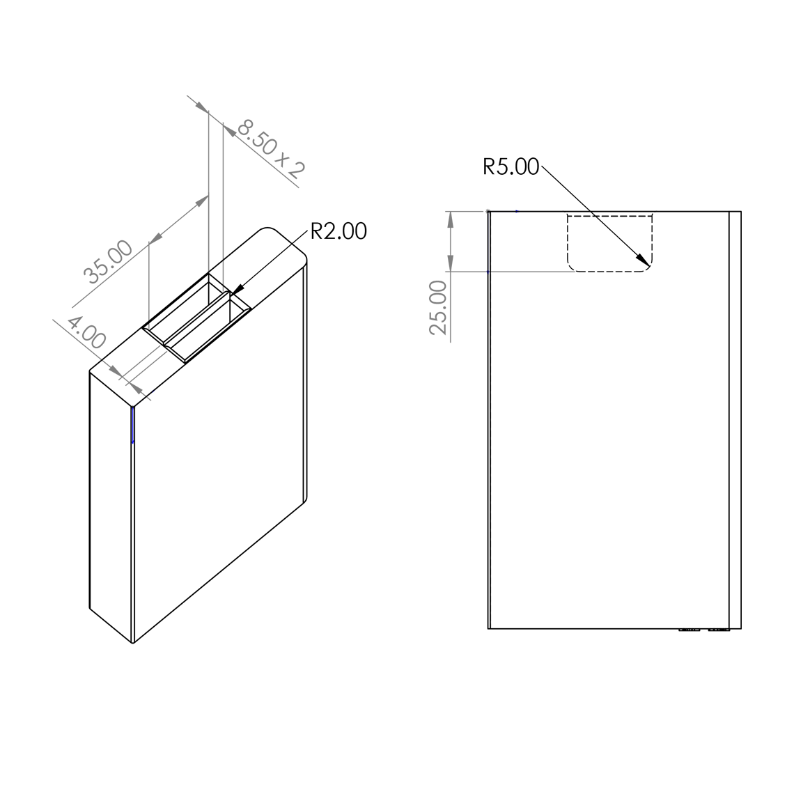 |
| a) Wireless phone b) Book | |
