# Supplementary material for: Modification of the toronto rehabilitation institute—hand function test for integration into robot-assisted therapy: technical validation and usability
Source: Biomed Eng Online. 2025 May 7;24:54. doi: 10.1186/s12938-025-01384-7 (PMC12060526; doi:10.1186/s12938-025-01384-7)
Supplement: Supplementary file 2 [file 12938_2025_1384_MOESM2_ESM.docx]

**APPENDIX B: Shelf Dimensions in mm**

| 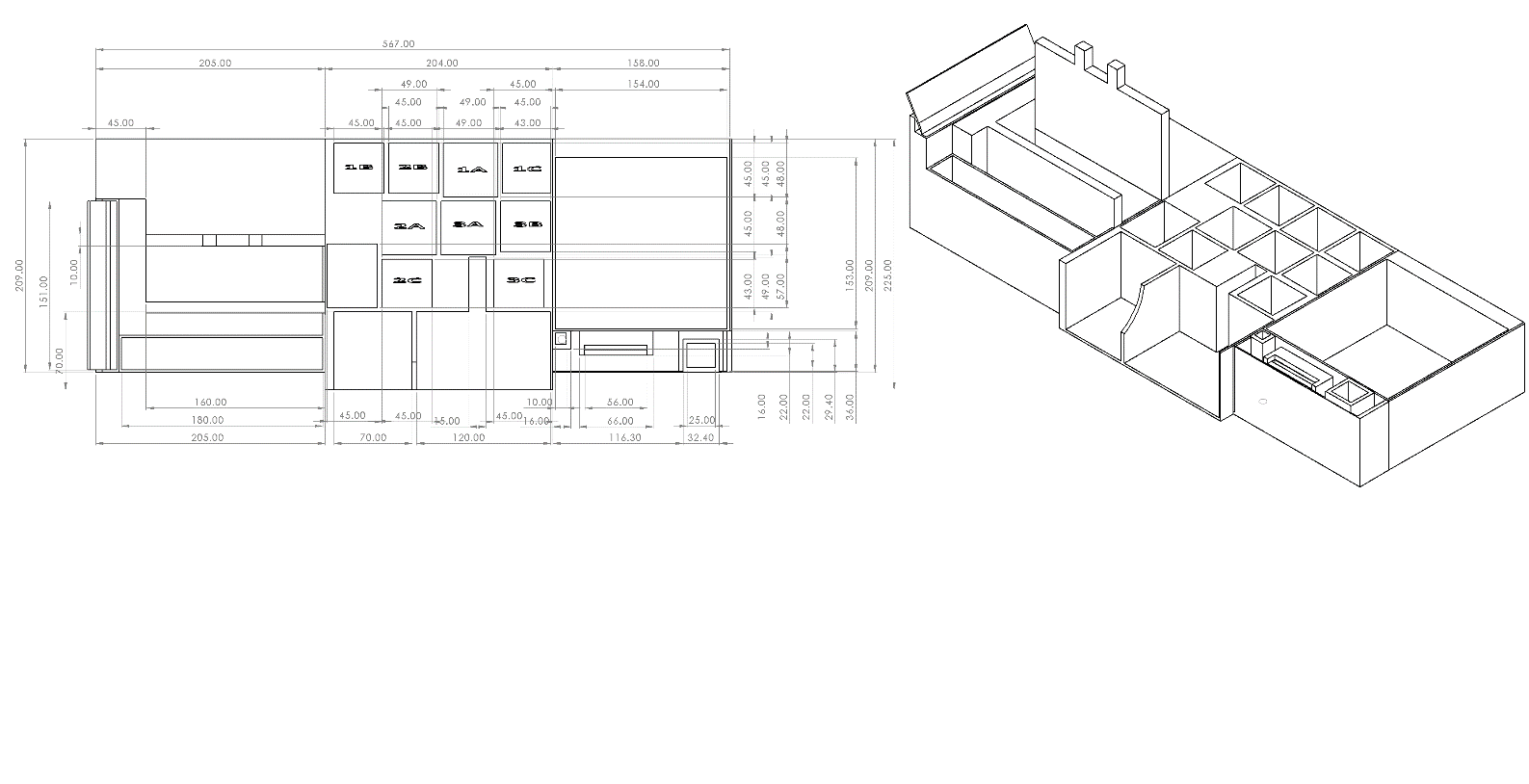 Top view of the shelf with dimensions in mm |
| --- |
| 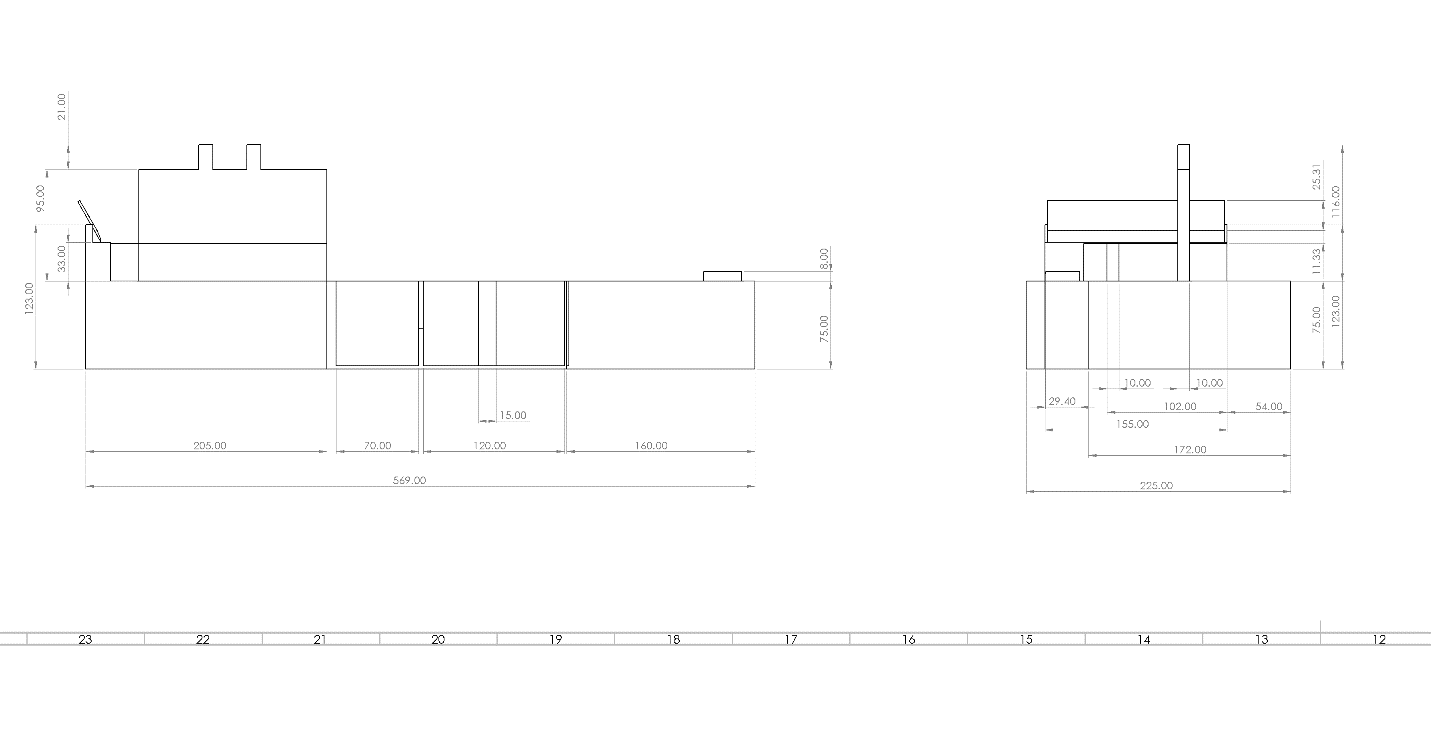 Front and side views of the shelf with dimensions in mm |

|  |
| --- |


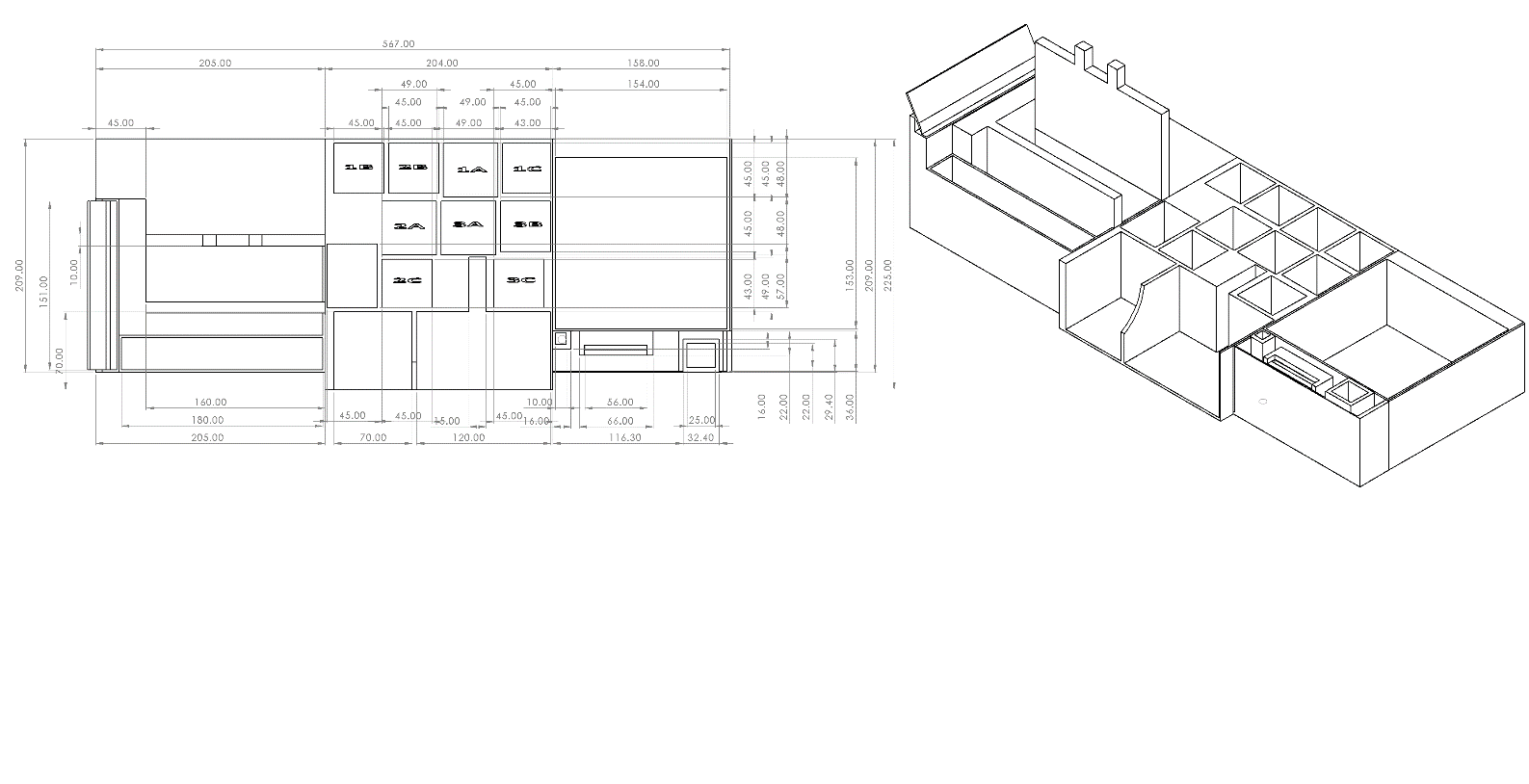


Isometric view of the shelf design
