# Supplementary figures and images for: Modification of the toronto rehabilitation institute—hand function test for integration into robot-assisted therapy: technical validation and usability
Source: Biomed Eng Online. 2025 May 7;24:54. doi: 10.1186/s12938-025-01384-7 (PMC12060526; doi:10.1186/s12938-025-01384-7)

| **APPENDIX C: Trajectory and Force Exertion During Extent-of-Reach Assessment** | |
| --- | --- |
| **P002**  **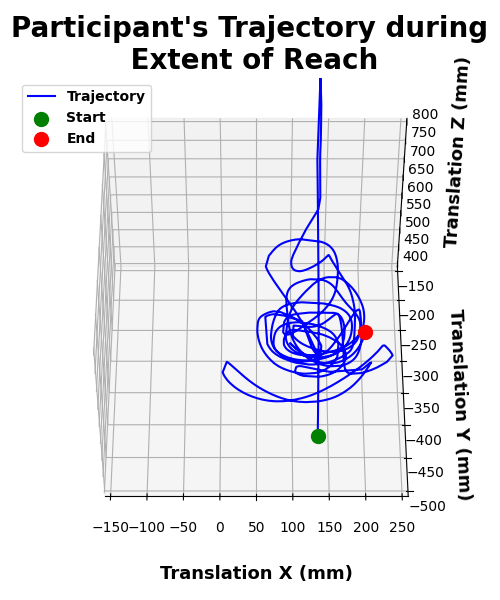** | **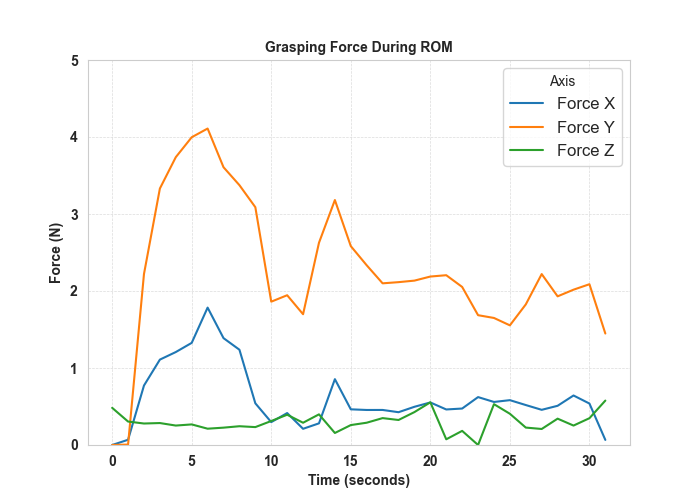** |
| **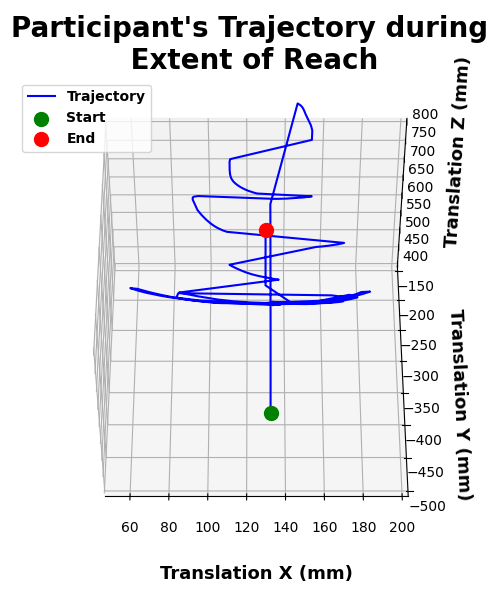P003** | **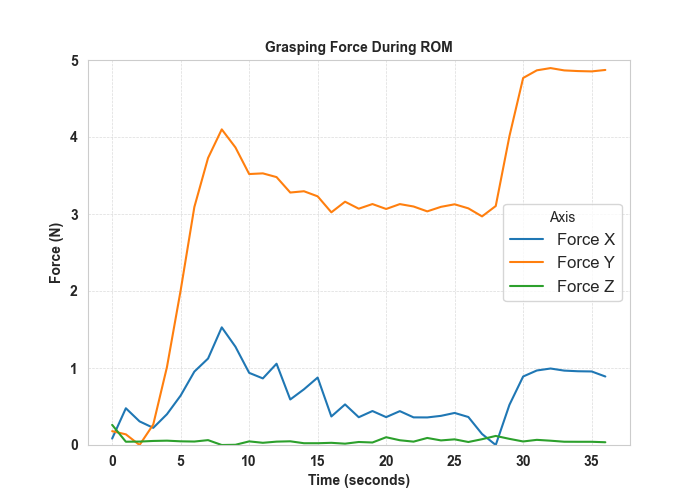** |
| **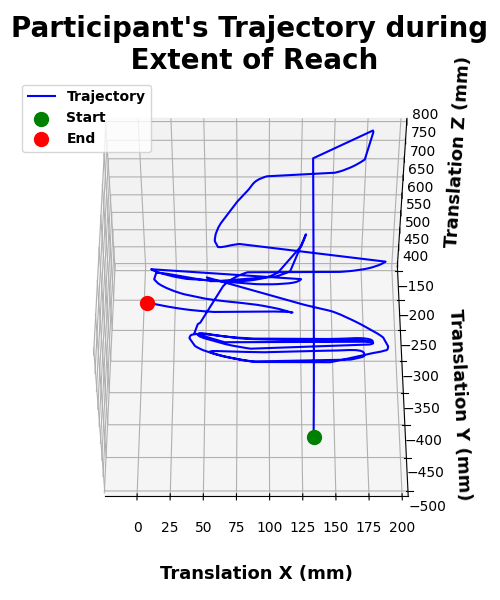P004** | **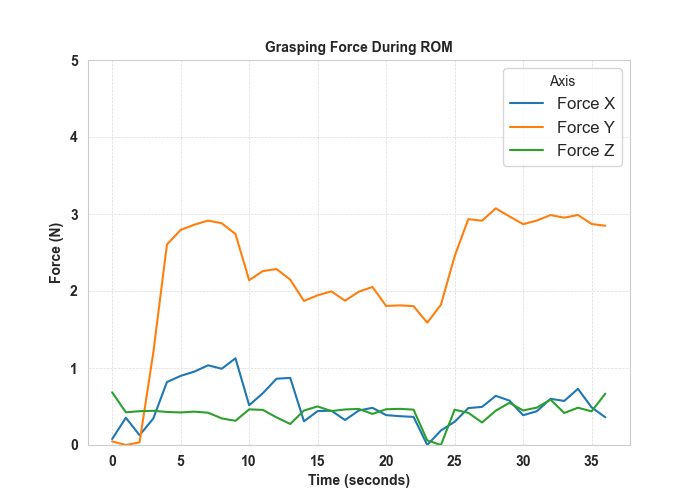** |
| **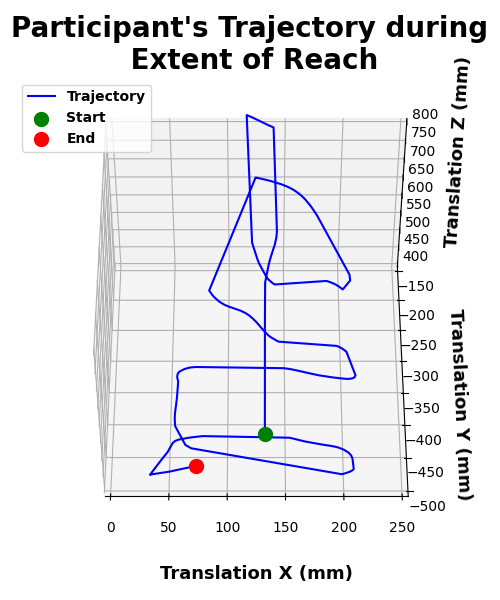P005** | **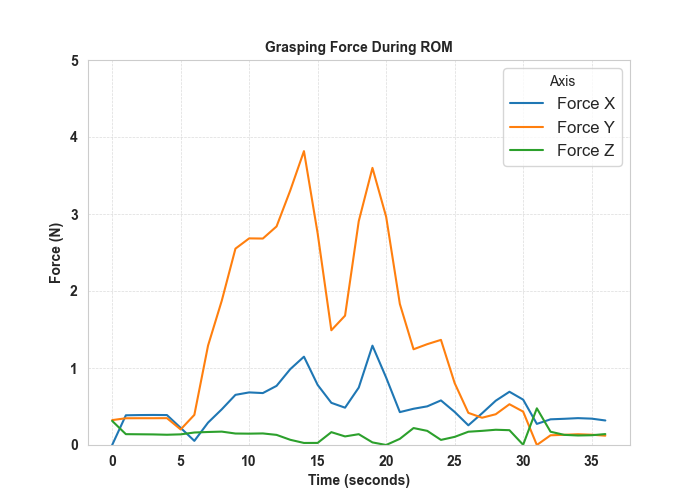** |

Supplement: Supplementary file 3 [file 12938_2025_1384_MOESM3_ESM.docx]
